# The correlation analysis of WeChat usage and depression among the middle-aged and elderly in China: the mediating role of social participation

**DOI:** 10.1186/s12889-023-15349-9

**Published:** 2023-03-10

**Authors:** Gaoling Wang, Jing Duan, Qianqian Kan, Yuqin Zhou, Zhaopeng Cheng, Shaoliang Tang

**Affiliations:** School of Health Economics and Management, Nanjing University of Chinese Medicine, Nanjing, China

**Keywords:** WeChat usage, Social participation, Depressive symptoms, The middle-aged and elderly

## Abstract

**Background:**

We aimed to explore the association between WeChat usage and depression in the Chinese middle-aged and elderly and the role of social participation.

**Methods:**

Data were obtained from China Health and Retirement Longitudinal Study (CHARLS) of 2018. The dependent variable was depressive symptoms, measured with the 10-item Center for Epidemiologic Studies Depression Scale (CES-D-10). We used the propensity score matching (PSM) to match the WeChat users with the non-WeChat users. Correlations between WeChat usage and depressive symptoms were verified by using logistic regression and linear regression, and the mediating role of social participation was verified by using stepwise regression and KHB method.

**Results:**

Four thousand five hundred forty-five samples were ultimately matched for analysis in this study. After including all control variables, results of logistic regression showed that WeChat usage was significantly associated with a lower prevalence of depression (aOR:0.701,95% CI: 0.605–0.812). And the results of linear regression showed that WeChat usage was associated with lower levels of depression which was significant (*p* < 0.001). The results of the stepwise regression and the KHB method showed a mediating role of social participation in WeChat usage and depressive symptoms. Among the four types of social participation, the mediating effect of recreational activities was significant, while the mediating effects of voluntary activities, cultural activities, and other activities were not significant. Meanwhile, the effect of WeChat usage on depression and the mediating effect of social participation were heterogeneous because of differences in age and gender.

**Conclusion:**

Social participation partly mediated the effect between WeChat usage and depression in middle-aged and older adults. Among the four types of social participation, only recreational activities had a mediating effect. Encouraging more active social participation and other types of social activities should be considered to improve the mental health of the middle-aged and older adults in China through social media usage.

**Supplementary Information:**

The online version contains supplementary material available at 10.1186/s12889-023-15349-9.

## Background

Depression affects mood, quality of life, and physical health [1], and is an independent risk factor for increased mortality [2, 3]. The prevalence of depression among Chinese adults was 20% from 2011 to 2019 [4]. Depression is one of the most common mental health problems among the middle-aged and elderly and has become an important public health problem in China [5]. It not only poses a significant threat to physical and mental health and ability to live, but also brings a heavy burden to families and society. With the aging and the popularity of healthy aging, the prevention and treatment of depression in the middle-aged and elderly is becoming increasingly important.

With the high popularity of the Internet, social media has become a factor that affects mental health. The use of social media could reduce anxiety and loneliness and promote physical health [6], as well as reduce depression levels and increase life satisfaction [7]. Social media can provide many convenient services such as information retrieval and online communication for the middle-aged and elderly with mobility problems, so it is gradually winning their affection. Online socialization could overcome geographical barriers [8], and keep older adults in close contact with family and friends [9], therefore enhancing their social support [10, 11]. WeChat is a popular social media in China and has become an inseparable part of the work and life [12]. According to a report by the Chinese Academy of Social Sciences, WeChat has become the most commonly used online social tool among the elderly [13]. WeChat usage has some positive effects on both physical and mental health of middle-aged and older adults. WeChat usage could reduce the risk of depression in the elderly [14] and had a significant positive impact on subjective health status [15]. A study suggested that WeChat usage may also boost memory in older adults by reducing risk of depression [16]. However, some studies have noted that excessive use of social media, such as WeChat addiction, can have negative effects on the physical, psychological, and social health of users [17–19]. We attempted to clarify the correlation between WeChat usage and depression among Chinese middle-aged and older adults, and to explore whether there were mediating variables in the correlation.

Social participation is an important factor, among influences on depression. The definition of social participation is not entirely uniform. In general, social participation refers to activities in which individuals engage in interactions with others in society or the community [20]. In China, whatever way the elderly keep in touch with society is considered to be a form of social participation [21].

Studies showed that social participation was an important part of healthy aging [22], and great for reducing loneliness, relieving depressive symptoms [23, 24], increasing life satisfaction [25], and improving mental health [26]. The higher the frequency and levels of social participation, the lower the risk of depression in older adults [27]. Social participation had a positive effect on health by increasing their social capital and contact between friends and family [28]. Older adults who engaged in social participation tend to have better cognitive behaviors [29]. Many types of socially participating activities had effects on mental health, such as volunteering and healthy exercise [30], paid work [31], religious activity [32]. Different types of social participation could positively affect physical and mental health as well as life satisfaction of older adults through different mechanisms [33].

In the digital era, it has been suggested that social media usage could contribute to social participation among the middle-aged and elderly [34]. Does social participation play a mediating role between WeChat usage and depression in middle-aged and older populations? Do different types of social participation have different mediating effects? In this study, we aimed to 1) clarify the relationship between WeChat usage, social participation, and depression in the middle-aged and elderly in China; and 2) explore the mediating role of different types of social participation activities in the relationship.

## Methods

### Study design and study sample

We used data from the China Health and Retirement Longitudinal Study (CHARLS) in wave 4 of 2018 for cross-sectional analysis. CHARLS aims to collect high-quality data on households and individuals aged 45 and older in China to analyze aging and promote research on healthy aging. CHARLS surveyed participants for basic information, health status, health insurance and health care, and retirement. CHARLS was approved by the Ethics Review Committee of Peking University, and all participants signed an informed consent form before the investigation and voluntarily participated in the survey [35].

**Fig. 1:**
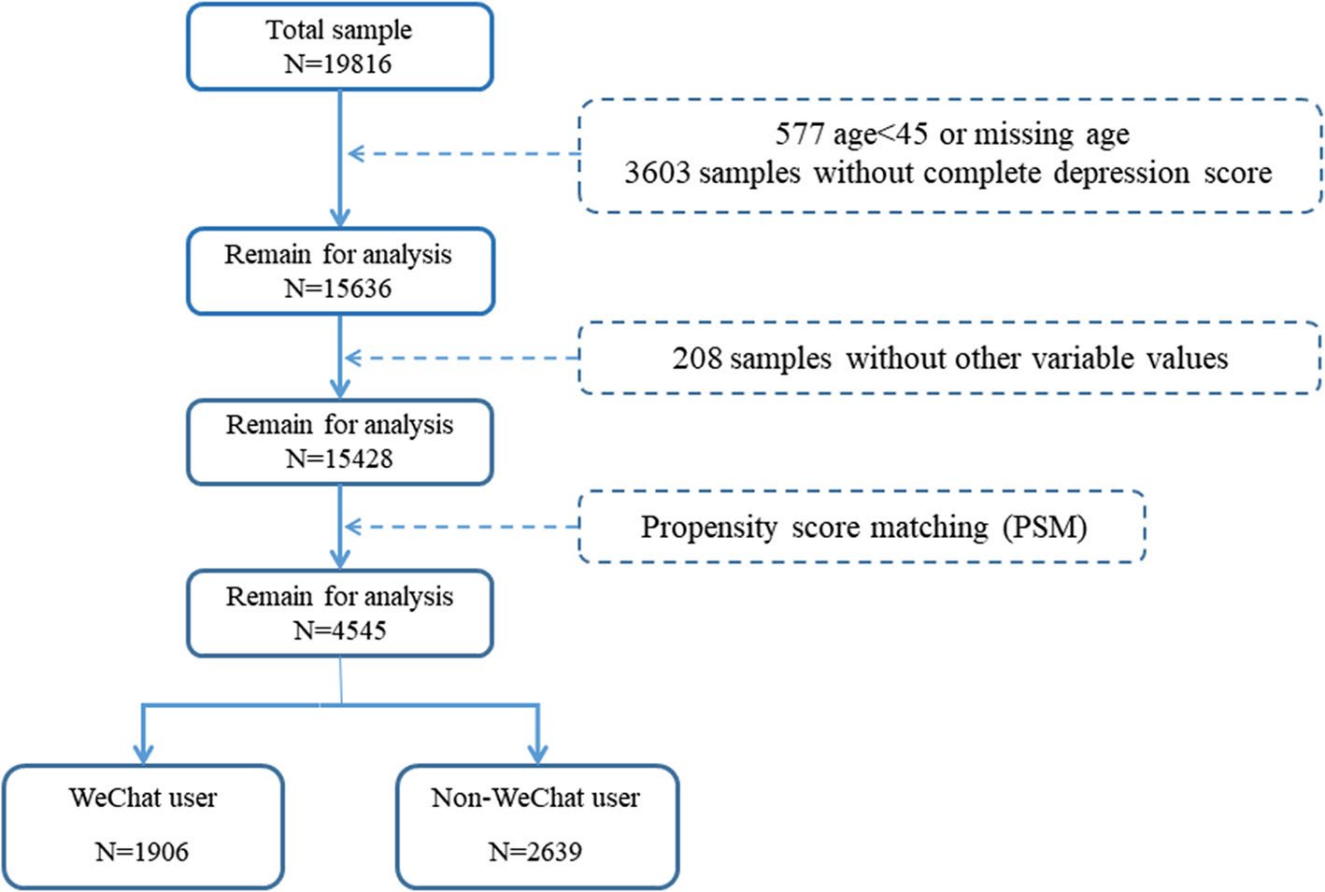
Data inclusion process

The target population selected was people aged 45 and above. The missing rate of the dependent variable values was 18.18%, and 15,636 participants were included in the study after excluding those with missing key variable values. The total missing rate for the remaining values of all variables was 1.33%, so we directly excluded participants with missing variable values, and the final number of samples included in the study was 15,428. Data inclusion process are shown in Fig. 1.

### Depression

The dependent variable in this study was depression, which was set as a dichotomous variable based on the CHARLS questionnaire. The 10-item Center for Epidemiologic Studies Depression Scale (CES-D-10), which is a simplified version of The Center for Epidemiologic Studies Depression Scale, is included in the questionnaire of the CHARLS. Each question was scored on a scale of 0–3 (rarely or not at all = 0, not too much = 1, sometimes or half the time = 2, most of the time = 3). The final score was calculated cumulatively, with a total score of 0–30. When the scores were ≥ 10, the respondent was considered to have depressive symptoms, and below 10, the respondent was considered normal. The depression score also reflected the levels of depression in the sample; therefore, the depression was also examined as a continuous variable in this study. The higher the depression score, the higher the levels of depression (Cronbach’s alpha = 0.805).

### WeChat usage

The independent variable of this study was WeChat usage status. In the questionnaire, participants were asked “Do you use WeChat?” and the answers included “yes” and “no”. When participants answered “yes”, they were considered to use WeChat, otherwise they were considered not to use WeChat.

### Social participation

The mediating variable in this study was social participation. Participants were asked in the questionnaire whether they had engaged in 10 activities in the past month. Participants were considered to be socially engaged if they participated in any of the activities. We also measured the levels of social participation. Participants earn one point for engaging in an activity. Points were accumulated, and the total score ranged from 0 to 10 points. The higher the score, the higher the levels of social participation.

We also tried to explore which type or types of social participation have a mediating role. Based on the experience of previous papers [29], we categorized the 10 activities mentioned in the questionnaire into voluntary activities, recreational activities, cultural activities, and other activities. Voluntary activities included: providing assistance to family, friends or neighbors who do not live with participants; doing volunteer or charitable work; caring for a sick or disabled adult who does not live with participants. Recreational activities included: Interacting with friends; playing Mahjong, chess, cards, or going to community clubs; going to sports, social or other types of clubs. Cultural activities included: participating in a community-related organization; participating in an educational or training course. Other activities contained: stock investment; and other activities. Participants were considered to have taken part in the type if they engaged in any of the activities in that type. Participants earn one point for engaging in an activity. Voluntary activities, recreational activities, cultural activities and other activities were respectively scored cumulatively to measure the participation levels in these four types of activities.

### Covariates

We selected a series of control variables that may be associated with depressive symptoms, including demographic characteristics [36–38] (age, gender, marital status, residence, education), health status and health behaviors [39–42] (self-reported health, activities of daily living scale (ADL), smoking, drinking, sleep duration, chronic disease status), and protective factors [31, 43–46] (health insurance, pension, employment status). For age, we selected people aged 45 and above; for marital status, we reclassified them according to the answers of the questionnaire, and considered married and living with spouse, married but not living with spouse temporarily as married; separated and no longer living with spouse, divorced, widowed, and never married as unmarried. Educational attainment was classified into five categories: no education, elementary school, middle school, high school, and college and above. Since the sleep time showed a skewed distribution, we logarithmically processed the sleep time. For chronic disease prevalence, we divided the population into five categories: no disease, one chronic disease, two chronic diseases, three chronic diseases, and four or more chronic diseases. The detailed coding of the variables is shown in Table 1.

**Table 1:** Coding of variables

Variable	Coding
Depression	< 10 = 0, ≥10 = 1
Levels of depression	0 ~ 30
WeChat usage	Not using the WeChat =0, Using the WeChat =1
Social participation	No = 0, Yes = 1
Levels of social participation	0 ~ 10
Voluntary activities	No = 0, Yes = 1
Levels of voluntary activities	No = 0, One kind = 1, Two kinds = 2, Three kinds = 3
Recreation	No = 0, Yes = 1
Levels of recreation	No = 0, One kind = 1, Two kinds = 2, Three kinds = 3
Cultural activities	No = 0, Yes = 1
Levels of cultural activities	No = 0, One kind = 1, Two kinds = 2
Other activities	No = 0, Yes = 1
Levels of other activities	No = 0, One kind = 1, Two kinds = 2
Age	≥45
Gender	Female = 0, Male =1
Marital status	Unmarried = 0, Married = 1
Residence	Rural = 1, Urban = 2
Education	No formal education = 1, Elementary school = 2, Middle school = 3, High school = 4, College or above = 5
Self-reported health	Very poor = 1, Poor = 2, Fair = 3, Good = 4, Very good = 5
ADL	No impaired = 0, Impaired = 1
Smoke status	Still have = 1, Quit = 2, No = 3
Drink status	No = 0, Yes = 1
Sleep time	Take the log of sleep time
Employment	No = 0, Yes = 1
Pension insurance	No = 0, Yes = 1
Medical insurance	No = 0, Yes = 1
Chronic diseases	No = 0, One kind = 1, Two kinds = 2, Three kinds = 3, Four kinds and more = 4

### Data analysis

We would use t-tests for continuous variables and chi-square tests for categorical variables to compare the sample characteristics between the WeChat users and non-WeChat users. Given that WeChat usage was not randomly distributed in the study, we used propensity score matching (PSM) to match the subjects. We used Psmatch2 to identify covariates and perform propensity score analysis. A 1:2 matching group was eventually constructed by nearest-neighbor matching, and the whole set of control variables was included in PSM.

Then, we used binary logistic regression and multiple linear regression on the matched groups to analyze the correlation between WeChat usage and depression. Since one of the dependent and mediating variables were both dichotomous, we used stepwise regression to examine the presence of a mediating effect. We also used the KHB method to check the mediating effect again and to analyze whether the mediating effect differed in different populations. The data were processed and analyzed using stata16.0.

## Results

**Fig. 2:**
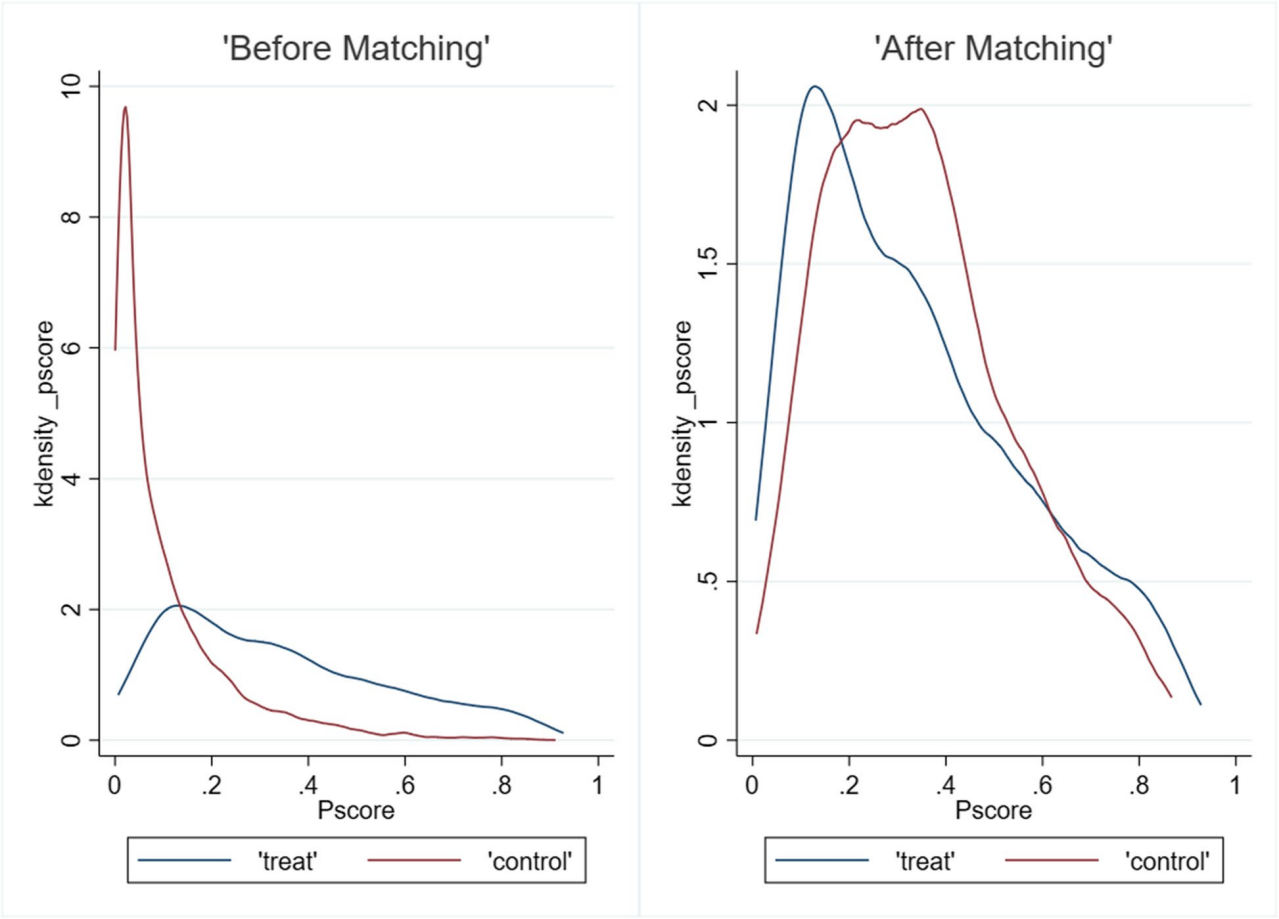
Kernel density map

After data cleaning, we included 15,428 participants in the study. After PSM, we finally matched 4545 participants for the final analysis, including 1906 participants who used WeChat and 2639 participants who did not use WeChat. The PSM results showed that the t-value of ATT was − 4.72, and the *p*-values of t-tests for all control variables were greater than 0.1, and the bias was less than 10%, which implied that the balance test was passed. According to the kernel density plot (Fig. 2), we found that the differences between the treated and control groups were large before matching, and the differences decreased significantly after matching, which indicated a better matching effect.

After PSM, the significance of differences in gender, marital status, sleep duration, ADL, smoking, employment, pension insurance, health insurance and chronic disease prevalence status no longer existed in the matched group, but the significance of differences between age, residence, education, self-reported health and drinking persisted. The mean age of all participants was 56.0 ± 7.3, the average age of WeChat users was 55.7 ± 7.4, and the average age of non-users was 56.2 ± 7.3. After matching, there were 2093 females (46.1%), 4229 were married (93.0%), 51.8% lived in rural areas, 70.7% had jobs, and only 26.4% did not suffer from chronic diseases. Among WeChat users, 451 (23.7%) had depression and their education levels were mostly concentrated in middle school (38.6%) and high school (27.1%), while 825 (31.3%) among non-WeChat users had depression and their education levels were mostly concentrated in elementary school (32.1%) and middle school (37.8%). The detailed characteristics of the other variables are shown in Table 2.

**Table 2:** Sample characteristics before and after PSM for WeChat users and non-WeChat users

Variable	Before PSM (***N*** = 15,428)			After PSM (***N*** = 4545)		
	WeChat user (***N*** = 2179)	Non-WeChat user (***N*** = 13,249)	***P*** value	WeChat user (***N*** = 1906)	Non-WeChat user (***N*** = 2639)	***P*** value
**Depressive symptom**			< 0.001			< 0.001
CES-D < 10	1680 (77.1)	8077 (61.0)		1455 (76.3)	1814 (68.7)	
CES-D > =10	499 (22.9)	5172 (39.0)		451 (23.7)	825 (31.3)	
**Levels of depression**	6.3 ± 0.1	8.8 ± 0.1	< 0.001	6.4 ± 0.1	7.5 ± 0.1	< 0.001
**Age**	55.3 ± 7.2	61.8 ± 9.3	< 0.001	55.7 ± 7.4	56.2 ± 7.3	0.033
**Gender**			< 0.001			0.558
Female	985 (45.2)	6887 (52.0)		868 (45.5)	1225 (46.4)	
Male	1194 (54.8)	6362 (48.0)		1038 (54.5)	1414 (53.6)	
**Marital status**			< 0.001			0.444
Unmarried	158 (7.3)	1754 (13.2)		139 (7.3)	177 (6.7)	
Married	2021 (92.7)	11,495 (86.8)		1767 (92.7)	2462 (93.3)	
**Residence**			< 0.001			< 0.001
Rural	937 (43.0)	9965 (75.2)		906 (47.5)	1450 (54.9)	
Urban	1242 (57.0)	3284 (24.8)		1000 (52.5)	1189 (45.1)	
**Education**			< 0.001			< 0.001
No formal education	33 (1.5)	2772 (20.9)		33 (1.7)	72 (2.7)	
Elementary school	506 (23.2)	6277 (47.4)		499 (26.2)	847 (32.1)	
Middle school	775 (35.6)	2926 (22.1)		736 (38.6)	997 (37.8)	
High school	657 (30.2)	1138 (8.6)		516 (27.1)	623 (23.6)	
College or above	208 (9.5)	136 (1.0)		122 (6.4)	100 (3.8)	
**Sleep time**	1.8 ± 0.3	1.8 ± 0.4	< 0.001	1.8 ± 0.3	1.8 ± 0.3	0.678
**Self-reported health**			< 0.001			0.049
Very poor	53 (2.4)	813 (5.8)		51 (2.7)	91 (3.4)	
Poor	206 (9.5)	2688 (20.3)		200 (10.5)	328 (12.4)	
Fair	1103 (50.6)	6580 (49.7)		986 (51.7)	1347 (51.0)	
Good	446 (20.5)	1564 (11.8)		357 (18.7)	431 (16.3)	
Very good	371 (17.0)	1604 (12.1)		312 (16.4)	442 (16.7)	
**ADL**			< 0.001			0.844
No impaired	2143 (98.3)	12,395 (93.6)		1870 (98.1)	2587 (98.0)	
Impaired	36 (1.7)	854 (6.4)		36 (1.9)	52 (2.0)	
**Smoke status**			0.235			0.501
Still have	643 (29.5)	3676 (27.7)		563 (29.5)	792 (30.0)	
Quit	309 (14.2)	1917 (14.5)		273 (14.3)	346 (13.1)	
No	1227 (56.3)	7656 (57.8)		1070 (56.1)	1501 (56.9)	
**Drink status**			< 0.001			0.042
No	1043 (47.9)	8845 (66.8)		966 (50.7)	1418 (53.7)	
Yes	1136 (52.1)	4404 (33.2)		940 (49.3)	1221 (46.3)	
**Employment**			< 0.001			0.418
No	629 (28.9)	4431 (33.4)		570 (29.9)	760 (28.8)	
Yes	1550 (71.1)	8818 (66.6)		1336 (70.1)	1879 (71.2)	
**Pension insurance**			< 0.001			0.602
No	164 (7.5)	1397 (10.5)		150 (7.9)	219 (8.3)	
Yes	2015 (92.5)	11,852 (89.5)		1756 (92.1)	2420 (91.7)	
**Medical insurance**			0.033			0.739
No	41 (1.9)	352 (2.6)		41 (2.2)	53 (2.0)	
Yes	2138 (98.1)	12,897 (97.3)		1865 (97.8)	2586 (98.0)	
**Chronic diseases**			< 0.001			0.348
No	597 (27.4)	2645 (20.0)		499 (26.2)	702 (26.6)	
One	566 (26.0)	3131 (23.6)		492 (25.8)	664 (25.2)	
Two	405 (18.6)	2722 (20.5)		357 (18.7)	516 (19.6)	
Three	292 (13.4)	1889 (14.3)		261 (13.7)	314 (11.9)	
Four and more	319 (14.6)	2862 (21.6)		297 (15.6)	443 (16.8)	

Regression results for WeChat usage, social participation and depression are presented in Tables 3 and 4. Table 3 used the occurrence of depression as the dependent variable. Model 1 measured WeChat usage and demographic characteristics, Model 2 included all control variables. Models 3 and 4 incorporated the presence and levels of social participation respectively. We could find that in models 1–4, WeChat usage was consistently and significantly associated with a lower risk of depression, as well as the presence and levels of social participation were significantly associated with the lower risk of depression. At the same time, we found that the effect of WeChat on depression was correspondingly reduced after including the variables related to social participation, which suggested social participation might have a partial mediating effect. We could also find that older age, being male, living in a city, having health insurance, better self-reported health and longer sleep duration were significantly associated with not having depression. In contrast, impaired ADL and having more chronic diseases were risk factors for depression.

**Table 3:** Binary logistic regression results of WeChat usage, social participation and depression

Variable	Model 1	Model 2	Model 3	Model 4
	OR (95%CI)	OR (95%CI)	OR (95%CI)	OR (95%CI)
**WeChat usage**	0.711^***^	0.701^***^	0.729^***^	0.731^***^
	(0.620–0.816)	(0.605–0.812)	(0.628–0.846)	(0.629–0.850)
**Social participation (Yes)**			0.792^**^	
			(0.683–0.919)	
**Levels of Social participation**				0.922^*^
				(0.866–0.982)
**Age**	0.987^**^	0.971^***^	0.970^***^	0.971^***^
	(0.978–0.997)	(0.960–0.983)	(0.959–0.982)	(0.959–0.982)
**Gender (Male)**	0.625^***^	0.694^**^	0.682^**^	0.683^**^
	(0.546–0.717)	(0.552–0.873)	(0.542–0.857)	(0.543–0.859)
**Marital status (Married)**	0.666^**^	0.784	0.782	0.784
	(0.518–0.855)	(0.597–1.030)	(0.595–1.028)	(0.597–1.031)
**Residence (Urban)**	0.770^***^	0.762^***^	0.759^***^	0.763^***^
	(0.668–0.887)	(0.649–0.894)	(0.646–0.890)	(0.650–0.896)
**Education**				
Elementary school	1.013	1.046	1.070	1.069
	(0.672–1.527)	(0.666–1.642)	(0.681–1.681)	(0.681–1.680)
Middle school	0.658^*^	0.724	0.752	0.750
	(0.436–0.993)	(0.461–1.138)	(0.478–1.183)	(0.477–1.180)
High school	0.524^**^	0.595^*^	0.622^*^	0.623^*^
	(0.342–0.803)	(0.372–0.949)	(0.389–0.994)	(0.389–0.996)
College or above	0.585^*^	0.705	0.752	0.764
	(0.346–0.992)	(0.398–1.250)	(0.423–1.336)	(0.429–1.360)
**Self-reported health**				
Poor		0.648^*^	0.641^*^	0.649^*^
		(0.429–0.979)	(0.424–0.968)	(0.430–0.981)
Fair		0.311^***^	0.310^***^	0.315^***^
		(0.211–0.459)	(0.210–0.457)	(0.213–0.464)
Good		0.184^***^	0.186^***^	0.189^***^
		(0.119–0.283)	(0.121–0.287)	(0.122–0.291)
Very good		0.120^***^	0.121^***^	0.123^***^
		(0.077–0.188)	(0.077–0.189)	(0.078–0.193)
**ADL (Impaired)**		2.693^***^	2.690^***^	2.660^***^
		(1.620–4.478)	(1.614–4.482)	(1.599–4.424)
**Smoke status**				
Quit		1.004	0.998	0.996
		(0.785–1.284)	(0.780–1.276)	(0.779–1.274)
No		1.058	1.048	1.047
		(0.841–1.331)	(0.832–1.318)	(0.832–1.317)
**Drink status (Yes)**		0.928	0.945	0.943
		(0.786–1.095)	(0.800–1.117)	(0.798–1.114)
**Sleep time**		0.297^***^	0.300^***^	0.300^***^
		(0.230–0.383)	(0.233–0.387)	(0.233–0.387)
**Employment (Yes)**		1.167	1.157	1.164
		(0.963–1.414)	(0.955–1.402)	(0.960–1.410)
**Pension insurance (Yes)**		0.991	0.999	0.999
		(0.762–1.289)	(0.768–1.300)	(0.768–1.299)
**Medical insurance (Yes)**		0.581^*^	0.588^*^	0.588^*^
		(0.355–0.949)	(0.360–0.961)	(0.360–0.960)
**Chronic diseases**				
One		1.128	1.135	1.136
		(0.911–1.395)	(0.917–1.405)	(0.918–1.406)
Two		1.299^*^	1.322^*^	1.320^*^
		(1.035–1.630)	(1.053–1.659)	(1.052–1.657)
Three		1.646^***^	1.689^***^	1.680^***^
		(1.276–2.123)	(1.308–2.181)	(1.302–2.169)
Four and more		2.000^***^	2.062^***^	2.074^***^
		(1.563–2.561)	(1.609–2.643)	(1.617–2.659)
**Constant**	2.661^**^	172.044^***^	187.642^***^	173.886^***^
	(1.296–5.464)	(54.026–547.865)	(58.778–599.020)	(54.568–554.111)
**N**	4545	4545	4545	4545

^*^ *p < 0.05,* ^**^ *p < 0.01,* ^***^ *p < 0.001*

**Table 4:** Regression results of WeChat usage, social participation and depression levels

Variable	Model 5	Model 6	Model 7	Model 8
**WeChat usage**	−0.873^***^	−0.787^***^	−0.716^***^	−0.692^***^
	(0.170)	(0.153)	(0.155)	(0.157)
**Social participation (Yes)**			−0.418^**^	
			(0.158)	
**Levels of Social participation**				−0.172^**^
				(0.063)
**Age**	−0.036^**^	− 0.071^***^	− 0.072^***^	− 0.072^***^
	(0.012)	(0.012)	(0.012)	(0.012)
**Gender (Male)**	−1.520^***^	−1.175^***^	−1.208^***^	−1.217^***^
	(0.173)	(0.237)	(0.237)	(0.238)
**Marital status (Married)**	−1.898^***^	− 1.220^***^	− 1.221^***^	− 1.212^***^
	(0.332)	(0.299)	(0.299)	(0.299)
**Residence (Urban)**	−0.775^***^	−0.689^***^	− 0.695^***^	− 0.682^***^
	(0.178)	(0.168)	(0.168)	(0.168)
**Education**				
Elementary school	−0.240	− 0.165	− 0.125	− 0.116
	(0.573)	(0.513)	(0.513)	(0.513)
Middle school	−1.495^**^	−1.029^*^	−0.962	−0.950
	(0.571)	(0.513)	(0.513)	(0.513)
High school	−2.270^***^	−1.701^**^	−1.621^**^	− 1.596^**^
	(0.584)	(0.525)	(0.525)	(0.526)
College or above	−2.499^***^	−1.820^**^	−1.712^**^	− 1.646^**^
	(0.683)	(0.614)	(0.615)	(0.617)
**Self-reported health**				
Poor		−2.662^***^	− 2.682^***^	− 2.662^***^
		(0.483)	(0.482)	(0.482)
Fair		−5.230^***^	−5.234^***^	−5.206^***^
		(0.454)	(0.454)	(0.454)
Good		−6.485^***^	−6.458^***^	−6.426^***^
		(0.487)	(0.487)	(0.487)
Very good		−7.633^***^	−7.616^***^	−7.576^***^
		(0.490)	(0.490)	(0.491)
**ADL (Impaired)**		2.472^***^	2.457^***^	2.438^***^
		(0.565)	(0.565)	(0.565)
**Smoke status**				
Quit		−0.405	−0.417	−0.421
		(0.249)	(0.249)	(0.249)
No		−0.141	−0.157	− 0.160
		(0.233)	(0.233)	(0.233)
**Drink status (Yes)**		−0.201	− 0.165	− 0.160
		(0.174)	(0.174)	(0.174)
**Sleep time**		−3.814^***^	−3.797^***^	−3.796^***^
		(0.272)	(0.272)	(0.272)
**Employment (Yes)**		0.428^*^	0.411^*^	0.423^*^
		(0.202)	(0.201)	(0.201)
**Pension insurance (Yes)**		0.073	0.085	0.088
		(0.285)	(0.285)	(0.285)
**Medical insurance (Yes)**		−1.512^**^	−1.486^**^	−1.479^**^
		(0.542)	(0.542)	(0.542)
**Chronic diseases**				
One		0.248	0.265	0.269
		(0.211)	(0.211)	(0.211)
Two		0.578^*^	0.608^**^	0.611^**^
		(0.234)	(0.234)	(0.234)
Three		1.066^***^	1.112^***^	1.112^***^
		(0.272)	(0.272)	(0.272)
Four and more		1.866^***^	1.918^***^	1.947^***^
		(0.268)	(0.269)	(0.270)
**Constant**	13.698^***^	27.387^***^	27.528^***^	27.382^***^
	(0.930)	(1.233)	(1.233)	(1.232)
**N**	4545	4545	4545	4545

*Note:* Standard errors in parentheses; ^*^ *p < 0.05,* ^**^ *p < 0.01,* ^***^ *p < 0.001*

In Table 4, we replaced the dependent variable with the levels of depression, and WeChat usage was consistently and significantly associated with lower levels of depression. In Models 7 and 8, the presence and levels of social participation were significantly associated with lower levels of depression. The effect of WeChat on the levels of depression was also reduced with the inclusion of variables related to social participation, suggesting that social participation may also have a partially mediating role. Compared to Table 3, the significance of the association between education levels and depression levels increased, and the association of being married and employed with lower depression levels also showed significance.

**Fig. 3:**
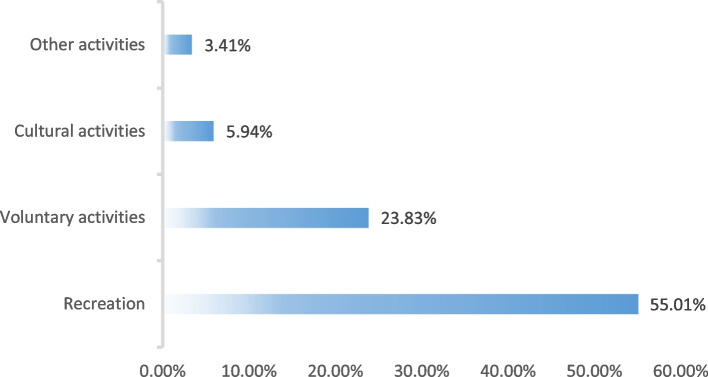
Participation rate of various activities in WeChat usage group

After matching, the most popular activity was recreational activities (55.01%), followed by voluntary activities (23.83%). While cultural activities (5.94%) and other activities (3.41%) had lower participation rates (Fig. 3). Table 5 reports the results of the binary logistic regression of the four types of social activities and depression after the inclusion of all control variables. The results showed that participating in recreational activities (aOR:0.812,95%CI:0.701–0.940) and participating in two recreational activities (aOR:0.860,95%CI:0.783–0.945) were significantly associated with non-depression. Also, in Table 5, WeChat usage continued to be significantly associated with non-having depression, and the effect of WeChat usage on depression decreased in all models compared to model 3. In Table 6, we replaced the dependent variable with the levels of depression. We found that participating in recreational activities (*p* < 0.05) and levels of recreational activities (*p* < 0.01) were significantly associated with lower levels of depression. Compared to Model 6, the effect of WeChat usage on depression levels decreased both in Model 18 and Model 22. Thus, there might be a partial mediating effect of recreational activities.

**Table 5:** Binary logistic regression results of different types of social participation and depression

Variable	Model 9	Model 10	Model 11	Model 12	Model 13	Model 14	Model 15	Model 16
**WeChat usage**	0.703^***^	0.726^***^	0.711^***^	0.706^***^	0.701^***^	0.734^***^	0.711^***^	0.705^***^
	(0.606–0.816)	(0.625–0.843)	(0.613–0.824)	(0.609–0.818)	(0.604–0.813)	(0.631–0.852)	(0.614–0.825)	(0.608–0.817)
**Voluntary activities (Yes)**	0.975							
	(0.820–1.159)							
**Recreation (Yes)**		0.812^**^						
		(0.701–0.940)						
**Cultural activities (Yes)**			0.718					
			(0.506–1.017)					
**Other activities (Yes)**				0.792				
				(0.504–1.244)				
**Levels of voluntary activities**								
One					0.941			
					(0.782–1.133)			
Two					1.134			
					(0.762–1.688)			
Three					1.400			
					(0.555–3.535)			
**Levels of recreation**								
One						0.859		
						(0.733–1.007)		
Two						0.697^**^		
						(0.557–0.873)		
Three						0.788		
						(0.483–1.284)		
**Levels of Cultural activities**								
One							0.746	
							(0.522–1.067)	
Two							0.395	
							(0.088–1.765)	
**Levels of other activities**								
One								0.764
								(0.483–1.208)
Two								6.116
								(0.310–120.763)
**Constant**	172.835^***^	180.163^***^	165.791^***^	169.944^***^	174.239^***^	179.866^***^	166.274^***^	170.102^***^
	(54.249–550.642)	(56.502–574.465)	(52.058–528.000)	(53.340–541.447)	(54.668–555.334)	(56.295–574.685)	(52.210–529.536)	(53.380–542.052)
**N**	4545	4545	4545	4545	4545	4545	4545	4545

*Note:* Control variables were included in all models; ^*^ *p < 0.05,* ^**^ *p < 0.01,* ^***^ *p < 0.001*

**Table 6:** Regression results of different types of social participation and the levels of depression

Variable	Model 17	Model 18	Model 19	Model 20	Model 21	Model 22	Model 23	Model 24
**WeChat usage**	− 0.757^***^	− 0.733^***^	−0.758^***^	− 0.769^***^	− 0.760^***^	−0.709^***^	−0.758^***^	−0.770^***^
	(0.155)	(0.155)	(0.154)	(0.154)	(0.155)	(0.156)	(0.154)	(0.154)
**Voluntary activities (Yes)**	−0.245							
	(0.180)							
**Recreation (Yes)**		−0.312^*^						
		(0.155)						
**Cultural activities (Yes)**			− 0.613					
			(0.325)					
**Other activities (Yes)**				−0.535				
				(0.420)				
**Levels of voluntary activities**								
One					−0.304			
					(0.192)			
Two					0.078			
					(0.413)			
Three					−0.110			
					(1.018)			
**Levels of recreation**								
One						−0.181		
						(0.169)		
Two						−0.616^**^		
						(0.227)		
Three						−0.525		
						(0.483)		
**Levels of Cultural activities**								
One							−0.602	
							(0.339)	
Two							−0.719	
							(1.000)	
**Levels of other activities**								
One								−0.558
								(0.422)
Two								1.294
								(3.575)
**Constant**	27.404^***^	27.458^***^	27.309^***^	27.349^***^	27.406^***^	27.414^***^	27.310^***^	27.350^***^
	(1.233)	(1.233)	(1.233)	(1.233)	(1.233)	(1.233)	(1.233)	(1.233)
**N**	4545	4545	4545	4545	4545	4545	4545	4545

*Note:* Standard errors in parentheses; Control variables were included in all models; ^*^ *p < 0.05,* ^**^ *p < 0.01,* ^***^ *p < 0.001*

Then we used binary logistic regression, ordered logistic regression and linear regression to test the significance of the correlation between the independent variables and the mediating variables. The results in Table 7 showed that the use of WeChat was significantly correlated with all four mediating variables. We therefore suggested that social participation and recreational activities might have a partially mediating role. Combining the results in Tables 3, 4, 5, 6, 7, we could calculate the percentage of mediating effects. When using being depressed as the ending variable, the mediating effect of performing social participation accounted for 49.86% of the total effect (a_1_b_1_ = − 0.177), the mediating effect of the levels of social participation accounted for 12.68% (a_2_b_2_ = − 0.045), the mediating effect of participating in recreational activities accounted for 42.82% (a_3_b_3_ = − 0.152). When the levels of depression were used as the ending variable, the mediating effect of performing social participation accounted for 40.28% of the total effect (a_4_b_4_ = − 0.317), the mediating effect of the levels of social participation accounted for 12.07% (a_5_b_5_ = − 0.095), the mediating effect of participating in recreational activities accounted for 28.97% (a_6_b_6_ = − 0.228). Since the level of recreational activities was an ordered categorical variable, we could not directly calculate the percentages.

**Table 7:** Regression results for WeChat usage and mediating variables

Variable	Model 25	Model 26	Model 27	Model 28
	Social participation OR (95%CI)	Level of social participation Coefficients (Standard error)	Recreation OR (95%CI)	Level of recreation OR (95%CI)
**WeChat usage**	2.137^***^	0.552^***^	2.078^***^	2.103^***^
	(1.880–2.429)	(0.036)	(1.836–2.353)	(1.877–2.356)
**Age**	0.991	−0.005	0.993	0.995
	(0.981–1.001)	(0.003)	(0.984–1.003)	(0.986–1.004)
**Gender (Male)**	0.698^***^	−0.246^***^	0.601^***^	0.589^***^
	(0.574–0.850)	(0.056)	(0.496–0.729)	(0.493–0.704)
**Marital status (Married)**	0.981	0.043	1.051	1.109
	(0.766–1.257)	(0.071)	(0.825–1.339)	(0.888–1.385)
**Residence (Urban)**	0.939	0.041	0.907	0.966
	(0.818–1.078)	(0.040)	(0.792–1.038)	(0.853–1.094)
**Education**				
Elementary school	1.497	0.286^*^	1.692^*^	1.695^*^
	(0.991–2.262)	(0.122)	(1.107–2.584)	(1.124–2.555)
Middle school	1.988^**^	0.463^***^	2.246^***^	2.137^***^
	(1.316–3.003)	(0.121)	(1.471–3.430)	(1.419–3.218)
High school	2.306^***^	0.613^***^	2.453^***^	2.458^***^
	(1.510–3.523)	(0.124)	(1.591–3.783)	(1.618–3.734)
College or above	3.233^***^	1.010^***^	3.343^***^	3.488^***^
	(1.930–5.415)	(0.145)	(2.003–5.579)	(2.160–5.632)
**Self-reported health**				
Poor	0.810	0.002	0.845	0.957
	(0.546–1.200)	(0.114)	(0.574–1.243)	(0.670–1.368)
Fair	0.964	0.141	0.994	1.105
	(0.665–1.397)	(0.107)	(0.691–1.431)	(0.792–1.543)
Good	1.351	0.341^**^	1.328	1.464^*^
	(0.905–2.017)	(0.115)	(0.897–1.965)	(1.024–2.094)
Very good	1.202	0.333^**^	1.284	1.442^*^
	(0.805–1.796)	(0.116)	(0.866–1.903)	(1.005–2.068)
**ADL (Impaired)**	0.846	− 0.203	0.833	0.746
	(0.535–1.337)	(0.134)	(0.529–1.312)	(0.489–1.138)
**Smoke status**				
Quit	0.879	−0.093	0.910	0.906
	(0.716–1.079)	(0.059)	(0.744–1.112)	(0.754–1.089)
No	0.838	−0.114^*^	0.773^**^	0.742^***^
	0.691–1.015)	(0.055)	(0.641–0.933)	(0.624–0.881)
**Drink status (Yes)**	1.493^***^	0.244^***^	1.508^***^	1.464^***^
	(1.293–1.726)	(0.041)	(1.309–1.737)	(1.286–1.666)
**Sleep time**	1.193	0.103	1.313^*^	1.291^*^
	(0.955–1.490)	(0.064)	(1.054–1.637)	(1.051–1.585)
**Employment (Yes)**	0.829^*^	− 0.029	0.815^*^	0.770^***^
	(0.702–0.980)	(0.048)	(0.692–0.959)	(0.662–0.894)
**Pension insurance (Yes)**	1.146	0.092	1.092	1.072
	(0.911–1.442)	(0.067)	(0.869–1.370)	(0.867–1.327)
**Medical insurance (Yes)**	1.308	0.189	1.243	1.292
	(0.847–2.019)	(0.128)	(0.805–1.919)	(0.860–1.942)
**Chronic diseases**				
One	1.197^*^	0.126^*^	1.146	1.178^*^
	(1.008–1.422)	(0.050)	(0.967–1.357)	(1.007–1.377)
Two	1.377^**^	0.193^***^	1.302^**^	1.294^**^
	(1.136–1.669)	(0.055)	(1.079–1.573)	(1.090–1.537)
Three	1.636^***^	0.267^***^	1.495^***^	1.504^***^
	(1.304–2.053)	(0.064)	(1.199–1.866)	(1.230–1.838)
Four and more	1.742^***^	0.474^***^	1.633^***^	1.745^***^
	(1.395–2.176)	(0.064)	(1.314–2.029)	(1.431–2.130)
**Constant**	0.509	−0.028	0.315^*^	
	(0.186–1.390)	(0.292)	(0.116–0.856)	
**N**	4545	4545	4545	4545

^*^ *p < 0.05,* ^**^ *p < 0.01,* ^***^ *p < 0.001*

To verify the accuracy of the mediating effect of social participation, we verified the mediating effect of social participation again using the KHB method, which extended the decomposability of linear models to nonlinear probability models for a variety of situations [47]. The results in Table 8 showed that the mediating effects of social participation and recreational activities were significant for the dependent variable whether it was occurrence of depression or levels of depression. We could assume that all four variables had mediating effects.

**Table 8:** KHB test for social participation

Effect	β	SE	***P***	95% CI		Mediation (%)	β	SE	***P***	95% CI		Mediation (%)
				Lower	Upper					Lower	Upper	
**WeChat usage--Social participation--Depression**							**WeChat usage--Social participation--Degree of depression**					
Total effect	− 0.356	0.075	0.000	− 0.503	− 0.209		− 0.787	0.153	0.000	−1.086	−0.487	
Direct effect	−0.317	0.076	0.000	−0.466	−0.167		−0.716	0.155	0.000	−1.020	−0.412	
Indirect effect	−0.040	0.013	0.003	−0.066	−0.014	11.11	−0.071	0.028	0.010	−0.125	−0.017	9.01
**WeChat usage--Levels of social participation--Depression**							**WeChat usage--Levels of social participation--Degree of depression**					
Total effect	−0.357	0.075	0.000	−0.505	−0.210		−0.787	0.153	0.000	−1.086	−0.487	
Direct effect	−0.313	0.077	0.000	−0.463	−0.162		−0.692	0.157	0.000	−0.999	−0.385	
Indirect effect	−0.045	0.018	0.013	−0.080	−0.009	12.49	−0.095	0.035	0.007	−0.164	−0.026	12.04
**WeChat usage--Recreation--Depression**							**WeChat usage--Recreation--Degree of depression**					
Total effect	−0.356	0.075	0.000	−0.503	−0.209		−0.787	0.153	0.000	−1.086	−0.487	
Direct effect	−0.320	0.076	0.000	−0.469	−0.171		−0.733	0.155	0.000	−1.037	−0.429	
Indirect effect	−0.036	0.013	0.007	−0.062	−0.010	10.08	−0.054	0.027	0.048	−0.107	−0.001	6.82
**WeChat usage--Levels of recreation--Depression**							**WeChat usage--Levels of recreation--Degree of depression**					
Total effect	−0.357	0.075	0.000	−0.504	−0.209		−0.787	0.153	0.000	−1.086	−0.487	
Direct effect	−0.310	0.076	0.000	−0.460	−0.160		−0.709	0.156	0.000	−1.014	−0.404	
Indirect effect	−0.047	0.015	0.002	−0.077	−0.017	13.17	− 0.078	0.030	0.010	−0.137	− 0.018	9.89

Finally, we divided the population into two groups respectively according to age and gender: the middle-aged (45–59), the elderly (60–84), the female and the male. We tried to analyze whether the correlation between WeChat usage and depression (see Supplementary Material S1–S2) as well as the mediating role of social engagement (see Supplementary Material S3, S4, S5, S6, S7, S8, S9, S10, S11, S12, S13) were heterogeneous because of age and gender. WeChat usage could reduce the levels and risk of depression in all groups and reduced the risk of depression better in the elderly than in the middle-aged. Apart from among women, social participation played the mediating role in almost all groups. However, the mediating effect of recreational activities was only present in the middle-aged.

## Discussion

This study concluded that WeChat usage was significantly associated with lower risk of depression and lower levels of depression in middle-aged and older adults, while social participation had mediating effects. Moreover, there was heterogeneity in the mediating effect across groups.

After we used PSM and adjusted for a range of control variables, the study found that WeChat usage was significantly associated with lower rates of depression and lower levels of depression. Social media usage was generally significantly associated with fewer depressive symptoms [48, 49], which is generally consistent with previous studies. There is an explanation that increasing age decreases physical functioning and impedes mobility in older adults, which increases the vulnerability to social isolation and loneliness dilemmas, causing mental health problems [50]. According to a survey, almost half of the elderly in China lived alone or only with their spouses [51]. Due to physical and distance reasons, it is difficult for them to meet with their relatives or friends frequently. Besides the convenience and speed of social media, its real-time updates and interactivity can facilitate the middle-aged and elderly to communicate with others and share their emotions, which may alleviate the problem of social isolation. For example, the use of social media such as WeChat and Facebook could expand the social network of the middle-aged and elderly, enhance their interaction with others, and promote communication and emotional cohesion with family members [52, 53]. So, we think that WeChat, as a comprehensive social media, offers online communication technology that may benefit people who cannot or rarely interact with others face-to-face, and its mental health benefits for middle-aged and older adults should be considered.

Social participation has a great impact on the occurrence of depression. We found that middle-aged and older adults who do not engage in social participation have a higher risk of depression than those who do, and among the four types of social participation activities proposed in this study, recreation could significantly reduce the probability and levels of depression. In line with the results of previous studies, active social participation could significantly reduce the risk of depression [22–24]. Because positive social participation may increase communication and interaction between middle-aged and older adults and help them understand the aging process as well as mitigate the negative effects of aging on mental health [54]. Recreational activities chosen according to their interests can also help them to relieve negative emotions effectively [21]. In this study, we found that recreation (socializing, playing Mahjong, playing chess, dancing, practicing qigong, etc.) was the main social activity of Chinese middle-aged and older adults. Competitive activities, such as playing chess and mahjong, can fully exercise their thinking skills and bring joy as well as a sense of accomplishment when they achieve victory. Leisure activities, such as dancing and qigong, can effectively relax their bodies. These activities are usually participated by many people and they can have sufficient conversations while engaging in recreational activities, which can allow them to relax and release the negative emotions caused by stressful events. Therefore, we hold the opinion that it may be effective to reduce the risk of depression by organizing more forms of recreational activities to meet the needs of the middle-aged and elderly, and encouraging them to try more forms of social participation.

This study found that WeChat usage can promote social participation in middle-aged and older adults, which is consistent with previous findings [34, 43, 55]. We further found that WeChat usage can influence depression through social participation. A possible reason is that online socializing can alleviate the shrinking social circle of older adults [15, 56]. Especially for those who have fewer social activities, online communication technologies allow them to gain more opportunities to communicate with others [57]. We think that the photo sharing, voice messaging and video calling technologies of social media have greatly increased the fun of communication for the middle-aged and elderly. It also fulfills their need for emotional expression and sharing their lives. Social media enhances their resistance to depression by increasing their emotional support and social support. On the other hand, social media can have an impact on different groups of people [58]. We think the online social function of social media is applicable not only to individuals, but also to groups. For example, different family WeChat groups can be established among different family members. The middle-aged and elderly can also form WeChat groups with like-minded people to promote further communication among them and facilitate them to organize more offline activities. Both online interaction and offline communication can alleviate the loneliness of the middle-aged and elderly and have a positive effect on mental health.

Our study found that WeChat usage promotes social participation among middle-aged and older adults, but only the recreational activities among them have a mediating effect. In previous studies, voluntary and cultural activities were also beneficial to physical and mental health [30, 46, 59], but in this study, we did not find that they could have a mediating effect. Probably the reason is that the sample of this study had a high participation rate in recreation and a low participation rate in other types of activities, especially cultural activities, which social activity participation status had an impact on the final results. Another possibility is that WeChat usage does not have an impact through these types of activities, which needs to be further investigated.

We also found some differences in the levels of effect of WeChat usage on depression and in the mediating effect of social participation because of differences in age and gender. The positive effect of WeChat usage on depression was better in the elderly than in the middle-aged. Although social participation had mediating effects in most groups, WeChat usage reduced the prevalence of depression through recreational activities only in middle-aged adults and men. Possible explanations are that communication is the most pressing need for WeChat usage [60]. The use of social media such as WeChat can help older adults communicate more easily with family and friends, share their lives and communicate emotionally [15], which can have a more positive effect on mental health. Compared with the elderly, middle-aged people are less likely to face obstacles in action and communication because of better physical health. On the other hand, older adults still face some difficulties in using WeChat, but the middle-aged group has better learning and receptive abilities, so they are more likely to fully utilize social media such as WeChat for recreational activities and thus gain mental health benefits. We propose that the accessibility of social media for older adults should be taken into account to fully utilize the positive effects of social media on mental health.

This study still has some limitations. First, we were using cross-sectional data as the CHARLS question item on WeChat first appeared in 2018, so we were unable to predict the causal relationship between WeChat usage and social participation and depression. In future studies, we will consider using longitudinal data to study this aspect; Second, the effect of WeChat usage on depression may also arise through other mediating variables, but this study only explored the mediating role of social participation. We will look for the possibility of other mediating variables in future studies. Our classification of activities may not be sufficiently objective because we did not find a unified classification standard for social participation activities. Finally, we have adjusted for covariates as much as possible and used propensity score matching to make the results as robust as possible, but the factors affecting depression are complex and there are still some unobserved or unaccounted for biases.

Despite some limitations, this study also has some practical implications. First, we verified that WeChat usage and social participation were protective factors for depression in Chinese middle-aged and elderly people. Second, we found that WeChat usage could reduce the risk and levels of depression through social participation. This would imply that we can further promote social participation rates through social media usage to reduce the risk of depression among the middle-aged and elderly.

## Conclusions

Our findings suggested that WeChat usage was significantly associated with lower risk of depression and lower levels of depression in a Chinese middle-aged and elderly population, with a partial mediating effect of social participation and recreational activities. These findings may have important implications for the prevention and treatment of mental health in middle-aged and older populations. Encouraging middle-aged and older adults to actively use social media and participate in social activities may be considered for the prevention and treatment of depression in the future to enhance mental health.

## Supplementary Information

**Additional file 1: S1.** Regression results of WeChat usage and whether depressed in different groups. **S2.** Regression results of WeChat usage and depression levels in different groups. **S3.** Regression results for social participation and depression in the middle-aged population. **S4.** Regression results for social participation and depression levels in the middle-aged population. **S5.** Regression results for social participation and depression in the elderly. **S6.** Regression results for social participation and depression levels in the elderly. **S7.** Regression results for social participation and depression in women. **S8.** Regression results for social participation and depression levels in women. **S9.** Regression results for social participation and depression in men. **S10.** Regression results for social participation and depression levels in men. **S11.** KHB test in the middle-aged. **S12.** KHB test in the elderly. **S13.** KHB test in men.

## Data Availability

The data used in this paper is from a publicly available database, and all data can be obtained through the official website of China Health and Retirement Longitudinal Study, http://charls.pku.edu.cn/.

## Abbreviations

CHARLS: China Health and Retirement Longitudinal Study
CES-D-10: The 10-item Center for Epidemiologic Studies Depression Scale
PSM: Propensity score matching
ADL: Activities of Daily Living Scale
