# Supplementary material for: The correlation analysis of WeChat usage and depression among the middle-aged and elderly in China: the mediating role of social participation
Source: BMC Public Health. 2023 Mar 10;23:462. doi: 10.1186/s12889-023-15349-9 (PMC9999613; doi:10.1186/s12889-023-15349-9)
Supplement: Supplementary file 1 — Additional file 1: S1. Regression results of WeChat usage and whether depressed in different groups. S2. Regression results of WeChat usage and depression levels in different groups. S3. Regression results for social participation and depression in the middle-aged population. S4. Regression results for social participation and depression levels in the middle-aged population. S5. Regression results for social participation and depression in the elderly. S6. Regression results for social participation and depression levels in the elderly. S7. Regression results for social participation and depression in women. S8. Regression results for social participation and depression levels in women. S9. Regression results for social participation and depression in men. S10. Regression results for social participation and depression levels in men. S11. KHB test in the middle-aged. S12. KHB test in the elderly. S13. KHB test in men. [file 12889_2023_15349_MOESM1_ESM.docx]

We found that in all groups, WeChat usage was significantly associated with a lower risk of depression, and the positive effect was greater for older adults than for middle-aged adults (S[1](#S1)). When we replaced the dependent variable with the levels of depression, WeChat usage was also consistently and significantly associated with lower levels of depression (S[2](#S2)).

We further introduced social participation into the regression analysis (S[3-10](#S3)) and used KHB to analyze the heterogeneity of mediating effects. We found that when using being depressed as the dependent variable, the levels of social participation, participation in recreational activities and the levels of recreational activities had mediating effects in the middle-aged population (S[11](#S11)); in the elderly (S[12](file:///C:\Users\14178\Desktop\新建%20DOCX%20文档.docx#S12)), there was a mediating role for engaging in social participation; in the female (S[7-8](#S7)), there was no mediating role for social participation; in the male (S[13](#S13)), mediating effects existed for engaging in social participation and recreational activities, as well as for the levels of recreational activities. When the levels of depression were used as dependent variables, no variables mediated the effect among middle-aged people (S[11](#S11)) and women (S[7-8](#S7)); engaging in social participation, levels of social participation and levels of recreational activities had mediating effects among older people (S[12](#S12)); engaging in social participation, engaging in recreational activities and levels of recreational activities had mediating effects among men (S[13](#S13)).

**S1** Regression results of WeChat usage and whether depressed in different groups

|  | **Model 1**  **OR (95%CI)** | **Model 2**  **OR (95%CI)** | **Model 3**  **OR (95%CI)** | **Model 4**  **OR (95%CI)** |
| --- | --- | --- | --- | --- |
| **Variable** | **45** | **60** | **Female** | **Male** |
| **WeChat usage** | 0.740^***^ | 0.560^***^ | 0.732^**^ | 0.673^***^ |
|  | (0.624-0.877) | (0.411-0.764) | (0.595-0.901) | (0.544-0.831) |
| **Age** | 0.993 | 0.972 | 0.965^***^ | 0.973^**^ |
|  | (0.969-1.017) | (0.940-1.005) | (0.949-0.982) | (0.957-0.990) |
| **Gender (Male)** | 0.620^***^ | 0.963 | - | - |
|  | (0.474-0.811) | (0.609-1.524) | - | - |
| **Marital status (Married)** | 0.592^**^ | 1.268 | 0.765 | 0.794 |
|  | (0.419-0.837) | (0.775-2.072) | (0.530-1.104) | (0.520-1.212) |
| **Residence (Urban)** | 0.733^***^ | 0.909 | 0.690^**^ | 0.829 |
|  | (0.610-0.881) | (0.644-1.283) | (0.547-0.870) | (0.662-1.037) |
| **Education** |  |  |  |  |
| Elementary school | 0.934 | 1.903 | 1.220 | 0.642 |
|  | (0.560-1.558) | (0.666-5.440) | (0.732-2.033) | (0.226-1.823) |
| Middle school | 0.679 | 1.032 | 0.834 | 0.457 |
|  | (0.407-1.135) | (0.358-2.972) | (0.498-1.396) | (0.162-1.294) |
| High school | 0.566^*^ | 0.757 | 0.688 | 0.384 |
|  | (0.331-0.970) | (0.261-2.196) | (0.396-1.194) | (0.134-1.098) |
| College or above | 0.861 | 0.646 | 0.672 | 0.485 |
|  | (0.443-1.675) | (0.189-2.215) | (0.306-1.477) | (0.158-1.494) |
| **Self-reported health** |  |  |  |  |
| Poor | 0.481^**^ | 1.279 | 0.721 | 0.599 |
|  | (0.286-0.809) | (0.608-2.692) | (0.387-1.344) | (0.343-1.046) |
| Fair | 0.239^***^ | 0.578 | 0.324^***^ | 0.319^***^ |
|  | (0.146-0.390) | (0.284-1.176) | (0.180-0.582) | (0.189-0.539) |
| Good | 0.140^***^ | 0.306^**^ | 0.225^***^ | 0.158^***^ |
|  | (0.082-0.239) | (0.133-0.704) | (0.119-0.427) | (0.087-0.286) |
| Very good | 0.099^***^ | 0.147^***^ | 0.142^***^ | 0.107^***^ |
|  | (0.057-0.171) | (0.059-0.370) | (0.073-0.276) | (0.057-0.197) |
| **ADL (Impaired)** | 3.716^***^ | 2.034 | 2.185^*^ | 3.242^**^ |
|  | (1.782-7.748) | (0.939-4.404) | (1.062-4.498) | (1.569-6.697) |
| **Smoke status** |  |  |  |  |
| Quit | 0.917 | 1.220 | 0.950 | 1.015 |
|  | (0.672-1.252) | (0.796-1.870) | (0.348-2.591) | (0.785-1.312) |
| No | 0.967 | 1.411 | 1.091 | 1.042 |
|  | (0.739-1.265) | (0.891-2.233) | (0.624-1.908) | (0.805-1.349) |
| **Drink status (Yes)** | 0.963 | 0.845 | 0.886 | 0.961 |
|  | (0.793-1.170) | (0.606-1.179) | (0.687-1.141) | (0.769-1.200) |
| **Sleep time** | 0.254^***^ | 0.377^***^ | 0.266^***^ | 0.323^***^ |
|  | (0.186-0.347) | (0.239-0.596) | (0.188-0.376) | (0.220-0.474) |
| **Employment (Yes)** | 1.005 | 1.518^*^ | 1.233 | 1.078 |
|  | (0.797-1.267) | (1.057-2.180) | (0.958-1.586) | (0.797-1.459) |
| **Pension insurance (Yes)** | 0.974 | 1.088 | 0.826 | 1.242 |
|  | (0.736-1.291) | (0.479-2.472) | (0.575-1.187) | (0.838-1.841) |
| **Medical insurance (Yes)** | 0.727 | 0.169^**^ | 0.933 | 0.382^**^ |
|  | (0.428-1.236) | (0.046-0.625) | (0.443-1.968) | (0.199-0.733) |
| **Chronic diseases** |  |  |  |  |
| One | 1.175 | 0.813 | 1.614^**^ | 0.765 |
|  | (0.931-1.484) | (0.470-1.406) | (1.201-2.170) | (0.561-1.042) |
| Two | 1.285 | 1.165 | 1.548^**^ | 1.071 |
|  | (0.994-1.660) | (0.691-1.963) | (1.128-2.124) | (0.773-1.484) |
| Three | 1.870^***^ | 0.966 | 2.150^***^ | 1.249 |
|  | (1.394-2.507) | (0.548-1.706) | (1.497-3.088) | (0.870-1.795) |
| Four and more | 1.825^***^ | 2.048^**^ | 2.987^***^ | 1.353 |
|  | (1.362-2.445) | (1.214-3.453) | (2.093-4.262) | (0.953-1.921) |
| **Constant** | 131.835^***^ | 58.816^*^ | 136.576^***^ | 232.728^***^ |
|  | (24.431-711.417) | (2.649-1305.772) | (25.809-722.732) | (37.198-1456.053) |
| **N** | 3253 | 1292 | 2093 | 2452 |
| **Experience p-value** | 0.041 | | 0.273 | |

*^*^ p < 0.05, ^**^ p < 0.01, ^***^ p < 0.001; Empirical p-values were used to test the significance of the differences in the coefficients of WeChat usage between the groups and were obtained by the bootstrapping method*

**S2** Regression results of WeChat usage and depression levels in different groups

|  | **Model 5** | **Model 6** | **Model 7** | **Model 8** |
| --- | --- | --- | --- | --- |
| **Variable** | **45** | **60** | **Female** | **Male** |
| **WeChat usage** | -0.650^***^ | -1.165^***^ | -0.954^***^ | -0.598^**^ |
|  | (0.182) | (0.281) | (0.241) | (0.195) |
| **Age** | -0.027 | -0.062^*^ | -0.099^***^ | -0.059^***^ |
|  | (0.026) | (0.030) | (0.019) | (0.016) |
| **Gender (Male)** | -1.658^***^ | -0.010 | - | - |
|  | (0.283) | (0.430) | - | - |
| **Marital status (Married)** | -2.001^***^ | -0.272 | -1.548^***^ | -1.045^*^ |
|  | (0.396) | (0.453) | (0.429) | (0.427) |
| **Residence (Urban)** | -0.817^***^ | -0.210 | -1.071^***^ | -0.374 |
|  | (0.196) | (0.334) | (0.272) | (0.210) |
| **Education** |  |  |  |  |
| Elementary school | -0.137 | 0.303 | 0.468 | -1.825 |
|  | (0.593) | (1.017) | (0.612) | (1.110) |
| Middle school | -0.959 | -0.766 | -0.524 | -2.436^*^ |
|  | (0.592) | (1.017) | (0.615) | (1.105) |
| High school | -1.462^*^ | -1.907 | -1.267 | -3.005^**^ |
|  | (0.615) | (1.019) | (0.648) | (1.112) |
| College or above | -1.159 | -2.342^*^ | -1.455 | -3.311^**^ |
|  | (0.740) | (1.134) | (0.869) | (1.170) |
| **Self-reported health** |  |  |  |  |
| Poor | -3.079^***^ | -1.201 | -1.986^**^ | -3.291^***^ |
|  | (0.581) | (0.857) | (0.744) | (0.629) |
| Fair | -5.711^***^ | -3.512^***^ | -4.860^***^ | -5.356^***^ |
|  | (0.544) | (0.814) | (0.703) | (0.590) |
| Good | -6.981^***^ | -4.861^***^ | -6.006^***^ | -6.685^***^ |
|  | (0.582) | (0.881) | (0.758) | (0.630) |
| Very good | -8.065^***^ | -6.157^***^ | -7.321^***^ | -7.728^***^ |
|  | (0.585) | (0.893) | (0.768) | (0.633) |
| **ADL (Impaired)** | 3.832^***^ | 0.794 | 1.824^*^ | 3.054^***^ |
|  | (0.741) | (0.855) | (0.825) | (0.775) |
| **Smoke status** |  |  |  |  |
| Quit | -0.638^*^ | -0.036 | -0.904 | -0.429 |
|  | (0.322) | (0.394) | (1.170) | (0.243) |
| No | -0.459 | 0.656 | -0.823 | -0.081 |
|  | (0.277) | (0.424) | (0.662) | (0.240) |
| **Drink status (Yes)** | -0.145 | -0.282 | -0.390 | -0.038 |
|  | (0.210) | (0.307) | (0.291) | (0.212) |
| **Sleep time** | -4.196^***^ | -3.106^***^ | -4.370^***^ | -3.173^***^ |
|  | (0.333) | (0.463) | (0.394) | (0.379) |
| **Employment (Yes)** | 0.183 | 0.821^*^ | 0.709^*^ | 0.019 |
|  | (0.254) | (0.340) | (0.290) | (0.283) |
| **Pension insurance (Yes)** | 0.015 | 0.258 | -0.528 | 0.601 |
|  | (0.307) | (0.796) | (0.440) | (0.370) |
| **Medical insurance (Yes)** | -0.745 | -5.501^***^ | -0.357 | -2.356^***^ |
|  | (0.584) | (1.496) | (0.877) | (0.682) |
| **Chronic diseases** |  |  |  |  |
| One | 0.388 | -0.454 | 0.885^**^ | -0.274 |
|  | (0.239) | (0.454) | (0.336) | (0.267) |
| Two | 0.544^*^ | 0.344 | 1.040^**^ | 0.210 |
|  | (0.274) | (0.459) | (0.362) | (0.303) |
| Three | 1.448^***^ | -0.092 | 1.859^***^ | 0.471 |
|  | (0.329) | (0.502) | (0.426) | (0.350) |
| Four and more | 1.812^***^ | 1.773^***^ | 2.926^***^ | 1.034^**^ |
|  | (0.330) | (0.487) | (0.421) | (0.346) |
| **Constant** | 26.744^***^ | 25.605^***^ | 28.978^***^ | 26.525^***^ |
|  | (1.810) | (3.019) | (1.907) | (1.826) |
| **N** | 3253 | 1292 | 2093 | 2452 |
| **Experience p-value** | 0.066 | | 0.125 | |

*Standard errors in parentheses；^*^ p < 0.05, ^**^ p < 0.01, ^***^ p < 0.001;* *Empirical p-values were used to test the significance of the differences in the coefficients of WeChat usage between the groups and were obtained by the bootstrapping method*

**S3** Regression results for social participation and depression in the middle-aged population

| **Variable** | (1) | (2) | (3) | (4) | (5) | (6) | (7) | (8) | (9) | (10) |
| --- | --- | --- | --- | --- | --- | --- | --- | --- | --- | --- |
| **WeChat usage** | 0.760^**^ | 0.768^**^ | 0.737^***^ | 0.763^**^ | 0.750^***^ | 0.745^***^ | 0.735^***^ | 0.774^**^ | 0.750^***^ | 0.743^***^ |
|  | (0.640-0.903) | (0.645-0.914) | (0.621-0.874) | (0.642-0.907) | (0.632-0.889) | (0.628-0.883) | (0.619-0.872) | (0.651-0.921) | (0.632-0.889) | (0.627-0.882) |
| **Social participation**  **(Yes)** | 0.838^*^ |  |  |  |  |  |  |  |  |  |
|  | (0.705-0.996) |  |  |  |  |  |  |  |  |  |
| **Levels of**  **Social participation** |  | 0.925^*^ |  |  |  |  |  |  |  |  |
|  |  | (0.860-0.995) |  |  |  |  |  |  |  |  |
| **Voluntary activities (Yes)** |  |  | 1.042 |  |  |  |  |  |  |  |
|  |  |  | (0.857-1.266) |  |  |  |  |  |  |  |
| **Recreation (Yes)** |  |  |  | 0.830^*^ |  |  |  |  |  |  |
|  |  |  |  | (0.700-0.984) |  |  |  |  |  |  |
| **Cultural activities**  **(Yes)** |  |  |  |  | 0.659 |  |  |  |  |  |
|  |  |  |  |  | (0.432-1.005) |  |  |  |  |  |
| **Other activities**  **(Yes)** |  |  |  |  |  | 0.737 |  |  |  |  |
|  |  |  |  |  |  | (0.422-1.289) |  |  |  |  |
| **Levels of voluntary activities** | | |  |  |  |  |  |  |  |  |
| One |  |  |  |  |  |  | 1.021 |  |  |  |
|  |  |  |  |  |  |  | (0.830-1.257) |  |  |  |
| Two |  |  |  |  |  |  | 1.131 |  |  |  |
|  |  |  |  |  |  |  | (0.721-1.773) |  |  |  |
| Three |  |  |  |  |  |  | 1.344 |  |  |  |
|  |  |  |  |  |  |  | (0.460-3.922) |  |  |  |
| **Levels of recreation** | |  |  |  |  |  |  |  |  |  |
| One |  |  |  |  |  |  |  | 0.892 |  |  |
|  |  |  |  |  |  |  |  | (0.742-1.073) |  |  |
| Two |  |  |  |  |  |  |  | 0.706^**^ |  |  |
|  |  |  |  |  |  |  |  | (0.543-0.919) |  |  |
| Three |  |  |  |  |  |  |  | 0.583 |  |  |
|  |  |  |  |  |  |  |  | (0.311-1.094) |  |  |
| **Levels of Cultural activities** | | |  |  |  |  |  |  |  |  |
| One |  |  |  |  |  |  |  |  | 0.662 |  |
|  |  |  |  |  |  |  |  |  | (0.428-1.024) |  |
| Two |  |  |  |  |  |  |  |  | 0.616 |  |
|  |  |  |  |  |  |  |  |  | (0.130-2.919) |  |
| **Levels of other activities** | |  |  |  |  |  |  |  |  |  |
| One |  |  |  |  |  |  |  |  |  | 0.696 |
|  |  |  |  |  |  |  |  |  |  | (0.393-1.232) |
| Two |  |  |  |  |  |  |  |  |  | 6.244 |
|  |  |  |  |  |  |  |  |  |  | (0.317-123.083) |
| **Constant** | 140.841^***^ | 133.102^***^ | 130.462^***^ | 134.596^***^ | 129.373^***^ | 131.532^***^ | 131.917^***^ | 129.913^***^ | 129.511^***^ | 132.527^***^ |
|  | (26.052-761.415) | (24.641-718.979) | (24.153-704.685) | (24.908-727.327) | (23.953-698.774) | (24.357-710.307) | (24.416-712.720) | (23.953-704.594) | (23.974-699.631) | (24.524-716.178) |
| N | 3253 | 3253 | 3253 | 3253 | 3253 | 3253 | 3253 | 3253 | 3253 | 3253 |

*Control variables were included in all models; ^*^ p < 0.05, ^**^ p < 0.01, ^***^ p < 0.001*

**S4** Regression results for social participation and depression levels in the middle-aged population

| **Variable** | (1) | (2) | (3) | (4) | (5) | (6) | (7) | (8) | (9) | (10) |
| --- | --- | --- | --- | --- | --- | --- | --- | --- | --- | --- |
| **WeChat usage** | -0.610^***^ | -0.575^**^ | -0.636^***^ | -0.612^***^ | -0.626^***^ | -0.631^***^ | -0.637^***^ | -0.587^**^ | -0.625^***^ | -0.632^***^ |
|  | (0.184) | (0.186) | (0.183) | (0.184) | (0.182) | (0.182) | (0.183) | (0.185) | (0.182) | (0.182) |
| **Social participation**  **(Yes)** | -0.254 |  |  |  |  |  |  |  |  |  |
|  | (0.188) |  |  |  |  |  |  |  |  |  |
| **Levels of**  **Social participation** |  | -0.147^*^ |  |  |  |  |  |  |  |  |
|  |  | (0.075) |  |  |  |  |  |  |  |  |
| **Voluntary activities (Yes)** |  |  | -0.134 |  |  |  |  |  |  |  |
|  |  |  | (0.209) |  |  |  |  |  |  |  |
| **Recreation (Yes)** |  |  |  | -0.222 |  |  |  |  |  |  |
|  |  |  |  | (0.185) |  |  |  |  |  |  |
| **Cultural activities**  **(Yes)** |  |  |  |  | -0.656 |  |  |  |  |  |
|  |  |  |  |  | (0.404) |  |  |  |  |  |
| **Other activities**  **(Yes)** |  |  |  |  |  | -0.681 |  |  |  |  |
|  |  |  |  |  |  | (0.531) |  |  |  |  |
| **Levels of voluntary activities** | | |  |  |  |  |  |  |  |  |
| One |  |  |  |  |  |  | -0.167 |  |  |  |
|  |  |  |  |  |  |  | (0.223) |  |  |  |
| Two |  |  |  |  |  |  | 0.074 |  |  |  |
|  |  |  |  |  |  |  | (0.482) |  |  |  |
| Three |  |  |  |  |  |  | -0.194 |  |  |  |
|  |  |  |  |  |  |  | (1.176) |  |  |  |
| **Levels of recreation** | |  |  |  |  |  |  |  |  |  |
| One |  |  |  |  |  |  |  | -0.093 |  |  |
|  |  |  |  |  |  |  |  | (0.201) |  |  |
| Two |  |  |  |  |  |  |  | -0.489 |  |  |
|  |  |  |  |  |  |  |  | (0.273) |  |  |
| Three |  |  |  |  |  |  |  | -0.759 |  |  |
|  |  |  |  |  |  |  |  | (0.598) |  |  |
| **Levels of Cultural activities** | | |  |  |  |  |  |  |  |  |
| One |  |  |  |  |  |  |  |  | -0.626 |  |
|  |  |  |  |  |  |  |  |  | (0.420) |  |
| Two |  |  |  |  |  |  |  |  | -0.977 |  |
|  |  |  |  |  |  |  |  |  | (1.283) |  |
| **Levels of other activities** | |  |  |  |  |  |  |  |  |  |
| One |  |  |  |  |  |  |  |  |  | -0.720 |
|  |  |  |  |  |  |  |  |  |  | (0.535) |
| Two |  |  |  |  |  |  |  |  |  | 1.229 |
|  |  |  |  |  |  |  |  |  |  | (3.600) |
| **Constant** | 26.844^***^ | 26.754^***^ | 26.776^***^ | 26.778^***^ | 26.711^***^ | 26.726^***^ | 26.785^***^ | 26.700^***^ | 26.716^***^ | 26.734^***^ |
|  | (1.811) | (1.809) | (1.811) | (1.810) | (1.810) | (1.810) | (1.812) | (1.813) | (1.810) | (1.810) |
| N | 3253 | 3253 | 3253 | 3253 | 3253 | 3253 | 3253 | 3253 | 3253 | 3253 |

*Standard errors in parentheses; Control variables were included in all models; ^*^ p < 0.05, ^**^ p < 0.01, ^***^ p < 0.001*

**S5** Regression results for social participation and depression in the elderly

| **Variable** | (1) | (2) | (3) | (4) | (5) | (6) | (7) | (8) | (9) | (10) |
| --- | --- | --- | --- | --- | --- | --- | --- | --- | --- | --- |
| **WeChat usage** | 0.603^**^ | 0.591^**^ | 0.580^***^ | 0.589^***^ | 0.565^***^ | 0.560^***^ | 0.572^***^ | 0.579^***^ | 0.567^***^ | 0.560^***^ |
|  | (0.439-0.827) | (0.429-0.814) | (0.423-0.796) | (0.430-0.807) | (0.413-0.773) | (0.410-0.765) | (0.416-0.786) | (0.421-0.796) | (0.414-0.775) | (0.410-0.765) |
| **Social participation**  **(Yes)** | 0.662^**^ |  |  |  |  |  |  |  |  |  |
|  | (0.491-0.891) |  |  |  |  |  |  |  |  |  |
| **Levels of**  **Social participation** |  | 0.919 |  |  |  |  |  |  |  |  |
|  |  | (0.808-1.046) |  |  |  |  |  |  |  |  |
| **Voluntary activities (Yes)** |  |  | 0.785 |  |  |  |  |  |  |  |
|  |  |  | (0.530-1.162) |  |  |  |  |  |  |  |
| **Recreation (Yes)** |  |  |  | 0.738^*^ |  |  |  |  |  |  |
|  |  |  |  | (0.549-0.991) |  |  |  |  |  |  |
| **Cultural activities**  **(Yes)** |  |  |  |  | 0.870 |  |  |  |  |  |
|  |  |  |  |  | (0.461-1.642) |  |  |  |  |  |
| **Other activities**  **(Yes)** |  |  |  |  |  | 1.004 |  |  |  |  |
|  |  |  |  |  |  | (0.463-2.177) |  |  |  |  |
| **Levels of voluntary activities** | | |  |  |  |  |  |  |  |  |
| One |  |  |  |  |  |  | 0.691 |  |  |  |
|  |  |  |  |  |  |  | (0.448-1.065) |  |  |  |
| Two |  |  |  |  |  |  | 1.250 |  |  |  |
|  |  |  |  |  |  |  | (0.530-2.947) |  |  |  |
| Three |  |  |  |  |  |  | 2.471 |  |  |  |
|  |  |  |  |  |  |  | (0.395-15.441) |  |  |  |
| **Levels of recreation** | |  |  |  |  |  |  |  |  |  |
| One |  |  |  |  |  |  |  | 0.736 |  |  |
|  |  |  |  |  |  |  |  | (0.531-1.022) |  |  |
| Two |  |  |  |  |  |  |  | 0.654 |  |  |
|  |  |  |  |  |  |  |  | (0.417-1.027) |  |  |
| Three |  |  |  |  |  |  |  | 1.382 |  |  |
|  |  |  |  |  |  |  |  | (0.614-3.111) |  |  |
| **Levels of Cultural activities** | | |  |  |  |  |  |  |  |  |
| One |  |  |  |  |  |  |  |  | 0.967 |  |
|  |  |  |  |  |  |  |  |  | (0.508-1.840) |  |
| Two |  |  |  |  |  |  |  |  | - |  |
|  |  |  |  |  |  |  |  |  | - |  |
| **Levels of other activities** | |  |  |  |  |  |  |  |  |  |
| One |  |  |  |  |  |  |  |  |  | 1.004 |
|  |  |  |  |  |  |  |  |  |  | (0.463-2.177) |
| Two |  |  |  |  |  |  |  |  |  | - |
|  |  |  |  |  |  |  |  |  |  | - |
| **Constant** | 69.280^**^ | 57.121 ^*^ | 58.890^*^ | 64.592^**^ | 56.661^*^ | 58.842^*^ | 62.214** | 71.618** | 59.937* | 58.842* |
|  | (3.096-1550.508) | (2.574-1267.723) | (2.655-1306.355) | (2.901-1437.989) | (2.542-1262.811) | (2.648-1307.719) | (2.798-1383.297) | (3.188-1608.991) | (2.684-1338.288) | (2.648-1307.719) |
| N | 1292 | 1292 | 1292 | 1292 | 1292 | 1292 | 1292 | 1292 | 1292 | 1292 |

*Control variables were included in all models; ^*^ p < 0.05, ^**^ p < 0.01, ^***^ p < 0.001*

**S6** Regression results for social participation and depression levels in the elderly

| **Variable** | (1) | (2) | (3) | (4) | (5) | (6) | (7) | (8) | (9) | (10) |
| --- | --- | --- | --- | --- | --- | --- | --- | --- | --- | --- |
| **WeChat usage** | -1.019^***^ | -1.014^***^ | -1.070^***^ | -1.071^***^ | -1.126^***^ | -1.153^***^ | -1.084^***^ | -1.049^***^ | -1.126^***^ | -1.153^***^ |
|  | (0.285) | (0.290) | (0.286) | (0.285) | (0.283) | (0.283) | (0.287) | (0.286) | (0.284) | (0.283) |
| **Social participation**  **(Yes)** | -0.783^**^ |  |  |  |  |  |  |  |  |  |
|  | (0.288) |  |  |  |  |  |  |  |  |  |
| **Levels of**  **Social participation** |  | -0.231^*^ |  |  |  |  |  |  |  |  |
|  |  | (0.114) |  |  |  |  |  |  |  |  |
| **Voluntary activities (Yes)** |  |  | -0.593 |  |  |  |  |  |  |  |
|  |  |  | (0.349) |  |  |  |  |  |  |  |
| **Recreation (Yes)** |  |  |  | -0.532 |  |  |  |  |  |  |
|  |  |  |  | (0.281) |  |  |  |  |  |  |
| **Cultural activities**  **(Yes)** |  |  |  |  | -0.547 |  |  |  |  |  |
|  |  |  |  |  | (0.536) |  |  |  |  |  |
| **Other activities**  **(Yes)** |  |  |  |  |  | -0.268 |  |  |  |  |
|  |  |  |  |  |  | (0.674) |  |  |  |  |
| **Levels of voluntary activities** | | |  |  |  |  |  |  |  |  |
| One |  |  |  |  |  |  | -0.743^*^ |  |  |  |
|  |  |  |  |  |  |  | (0.376) |  |  |  |
| Two |  |  |  |  |  |  | -0.009 |  |  |  |
|  |  |  |  |  |  |  | (0.792) |  |  |  |
| Three |  |  |  |  |  |  | 1.068 |  |  |  |
|  |  |  |  |  |  |  | (2.014) |  |  |  |
| **Levels of recreation** |  |  |  |  |  |  |  |  |  |  |
| One |  |  |  |  |  |  |  | -0.403 |  |  |
|  |  |  |  |  |  |  |  | (0.309) |  |  |
| Two |  |  |  |  |  |  |  | -0.911^*^ |  |  |
|  |  |  |  |  |  |  |  | (0.401) |  |  |
| Three |  |  |  |  |  |  |  | -0.094 |  |  |
|  |  |  |  |  |  |  |  | (0.804) |  |  |
| **Levels of Cultural activities** | | |  |  |  |  |  |  |  |  |
| One |  |  |  |  |  |  |  |  | -0.575 |  |
|  |  |  |  |  |  |  |  |  | (0.563) |  |
| Two |  |  |  |  |  |  |  |  | -0.311 |  |
|  |  |  |  |  |  |  |  |  | (1.558) |  |
| **Levels of other activities** | |  |  |  |  |  |  |  |  |  |
| One |  |  |  |  |  |  |  |  |  | -0.268 |
|  |  |  |  |  |  |  |  |  |  | (0.674) |
| Two |  |  |  |  |  |  |  |  |  | - |
|  |  |  |  |  |  |  |  |  |  | - |
| **Constant** | 25.839^***^ | 25.494^***^ | 25.520^***^ | 25.755^***^ | 25.451^***^ | 25.586^***^ | 25.572^***^ | 25.656^***^ | 25.435^***^ | 25.586^***^ |
|  | (3.013) | (3.016) | (3.017) | (3.017) | (3.023) | (3.020) | (3.019) | (3.019) | (3.025) | (3.020) |
| N | 1292 | 1292 | 1292 | 1292 | 1292 | 1292 | 1292 | 1292 | 1292 | 1292 |

*Standard errors in parentheses; Control variables were included in all models; ^*^ p < 0.05, ^**^ p < 0.01, ^***^ p < 0.001*

**S7** Regression results for social participation and depression in women

| **Variable** | (1) | (2) | (3) | (4) | (5) | (6) | (7) | (8) | (9) | (10) |
| --- | --- | --- | --- | --- | --- | --- | --- | --- | --- | --- |
| **WeChat usage** | 0.744^**^ | 0.754^**^ | 0.737^**^ | 0.739^**^ | 0.742^**^ | 0.740^**^ | 0.729^**^ | 0.750^**^ | 0.742^**^ | 0.740^**^ |
|  | (0.603-0.918) | (0.610-0.933) | (0.598-0.908) | (0.599-0.912) | (0.602-0.913) | (0.601-0.912) | (0.591-0.899) | (0.607-0.926) | (0.603-0.914) | (0.601-0.912) |
| **Social participation**  **(Yes)** | 0.897 |  |  |  |  |  |  |  |  |  |
|  | (0.727-1.108) |  |  |  |  |  |  |  |  |  |
| **Levels of**  **Social participation** |  | 0.940 |  |  |  |  |  |  |  |  |
|  |  | (0.860-1.028) |  |  |  |  |  |  |  |  |
| **Voluntary activities (Yes)** |  |  | 0.927 |  |  |  |  |  |  |  |
|  |  |  | (0.719-1.195) |  |  |  |  |  |  |  |
| **Recreation (Yes)** |  |  |  | 0.944 |  |  |  |  |  |  |
|  |  |  |  | (0.768-1.162) |  |  |  |  |  |  |
| **Cultural activities**  **(Yes)** |  |  |  |  | 0.618 |  |  |  |  |  |
|  |  |  |  |  | (0.362-1.055) |  |  |  |  |  |
| **Other activities**  **(Yes)** |  |  |  |  |  | 0.667 |  |  |  |  |
|  |  |  |  |  |  | (0.333-1.338) |  |  |  |  |
| **Levels of voluntary activities** | | |  |  |  |  |  |  |  |  |
| One |  |  |  |  |  |  | 0.910 |  |  |  |
|  |  |  |  |  |  |  | (0.693-1.193) |  |  |  |
| Two |  |  |  |  |  |  | 0.865 |  |  |  |
|  |  |  |  |  |  |  | (0.470-1.592) |  |  |  |
| Three |  |  |  |  |  |  | 2.587 |  |  |  |
|  |  |  |  |  |  |  | (0.719-9.309) |  |  |  |
| **Levels of recreation** | |  |  |  |  |  |  |  |  |  |
| One |  |  |  |  |  |  |  | 1.008 |  |  |
|  |  |  |  |  |  |  |  | (0.805-1.262) |  |  |
| Two |  |  |  |  |  |  |  | 0.787 |  |  |
|  |  |  |  |  |  |  |  | (0.574-1.080) |  |  |
| Three |  |  |  |  |  |  |  | 0.936 |  |  |
|  |  |  |  |  |  |  |  | (0.510-1.717) |  |  |
| **Levels of Cultural activities** | | |  |  |  |  |  |  |  |  |
| One |  |  |  |  |  |  |  |  | 0.663 |  |
|  |  |  |  |  |  |  |  |  | (0.387-1.136) |  |
| Two |  |  |  |  |  |  |  |  | - |  |
|  |  |  |  |  |  |  |  |  | - |  |
| **Levels of other activities** | |  |  |  |  |  |  |  |  |  |
| One |  |  |  |  |  |  |  |  |  | 0.670 |
|  |  |  |  |  |  |  |  |  |  | (0.334-1.345) |
| Two |  |  |  |  |  |  |  |  |  | - |
|  |  |  |  |  |  |  |  |  |  | - |
| **Constant** | 139.157^***^ | 133.554^***^ | 137.577^***^ | 136.639^***^ | 133.390^***^ | 133.629^***^ | 141.287^***^ | 137.445^***^ | 133.167^***^ | 133.413^***^ |
|  | (26.297-736.386) | (25.241-706.653) | (25.996-728.086) | (25.829-722.823) | (25.228-705.272) | (25.240-707.488) | (26.651-749.021) | (25.848-730.864) | (25.177-704.367) | (25.200-706.319) |
| N | 2093 | 2093 | 2093 | 2093 | 2093 | 2093 | 2093 | 2093 | 2093 | 2093 |

*Control variables were included in all models; ^*^ p < 0.05, ^**^ p < 0.01, ^***^ p < 0.001*

**S8** Regression results for social participation and depression levels in women

| **Variable** | (1) | (2) | (3) | (4) | (5) | (6) | (7) | (8) | (9) | (10) |
| --- | --- | --- | --- | --- | --- | --- | --- | --- | --- | --- |
| **WeChat usage** | -0.923^***^ | -0.868^***^ | -0.920^***^ | -0.952^***^ | -0.919^***^ | -0.911^***^ | -0.929^***^ | -0.906^***^ | -0.918^***^ | -0.912^***^ |
|  | (0.243) | (0.245) | (0.242) | (0.244) | (0.241) | (0.242) | (0.242) | (0.245) | (0.241) | (0.242) |
| **Social participation**  **(Yes)** | -0.212 |  |  |  |  |  |  |  |  |  |
|  | (0.248) |  |  |  |  |  |  |  |  |  |
| **Levels of**  **Social participation** |  | -0.171 |  |  |  |  |  |  |  |  |
|  |  | (0.098) |  |  |  |  |  |  |  |  |
| **Voluntary activities (Yes)** |  |  | -0.372 |  |  |  |  |  |  |  |
|  |  |  | (0.289) |  |  |  |  |  |  |  |
| **Recreation (Yes)** |  |  |  | -0.010 |  |  |  |  |  |  |
|  |  |  |  | (0.243) |  |  |  |  |  |  |
| **Cultural activities**  **(Yes)** |  |  |  |  | -1.014 |  |  |  |  |  |
|  |  |  |  |  | (0.523) |  |  |  |  |  |
| **Other activities**  **(Yes)** |  |  |  |  |  | -1.194 |  |  |  |  |
|  |  |  |  |  |  | (0.693) |  |  |  |  |
| **Levels of voluntary activities** | | |  |  |  |  |  |  |  |  |
| One |  |  |  |  |  |  | -0.398 |  |  |  |
|  |  |  |  |  |  |  | (0.309) |  |  |  |
| Two |  |  |  |  |  |  | -0.381 |  |  |  |
|  |  |  |  |  |  |  | (0.666) |  |  |  |
| Three |  |  |  |  |  |  | 0.706 |  |  |  |
|  |  |  |  |  |  |  | (1.632) |  |  |  |
| **Levels of recreation** | |  |  |  |  |  |  |  |  |  |
| One |  |  |  |  |  |  |  | 0.195 |  |  |
|  |  |  |  |  |  |  |  | (0.265) |  |  |
| Two |  |  |  |  |  |  |  | -0.531 |  |  |
|  |  |  |  |  |  |  |  | (0.358) |  |  |
| Three |  |  |  |  |  |  |  | -0.123 |  |  |
|  |  |  |  |  |  |  |  | (0.666) |  |  |
| **Levels of Cultural activities** | | |  |  |  |  |  |  |  |  |
| One |  |  |  |  |  |  |  |  | -0.852 |  |
|  |  |  |  |  |  |  |  |  | (0.540) |  |
| Two |  |  |  |  |  |  |  |  | -2.995 |  |
|  |  |  |  |  |  |  |  |  | (1.723) |  |
| **Levels of other activities** | |  |  |  |  |  |  |  |  |  |
| One |  |  |  |  |  |  |  |  |  | -1.212 |
|  |  |  |  |  |  |  |  |  |  | (0.698) |
| Two |  |  |  |  |  |  |  |  |  | -0.044 |
|  |  |  |  |  |  |  |  |  |  | (5.366) |
| **Constant** | 29.025^***^ | 28.938^***^ | 28.986^***^ | 28.979^***^ | 28.908^***^ | 28.915^***^ | 29.003^***^ | 28.969^***^ | 28.914^***^ | 28.920^***^ |
|  | (1.908) | (1.907) | (1.907) | (1.908) | (1.907) | (1.907) | (1.908) | (1.909) | (1.906) | (1.908) |
| N | 2093 | 2093 | 2093 | 2093 | 2093 | 2093 | 2093 | 2093 | 2093 | 2093 |

*Standard errors in parentheses; Control variables were included in all models; ^*^ p < 0.05, ^**^ p < 0.01, ^***^ p < 0.001*

**S9** Regression results for social participation and depression in men

| **Variable** | (1) | (2) | (3) | (4) | (5) | (6) | (7) | (8) | (9) | (10) |
| --- | --- | --- | --- | --- | --- | --- | --- | --- | --- | --- |
| **WeChat usage** | 0.713^**^ | 0.705^**^ | 0.668^***^ | 0.713^**^ | 0.678^***^ | 0.674^***^ | 0.665^***^ | 0.718^**^ | 0.678^***^ | 0.672^***^ |
|  | (0.574-0.884) | (0.567-0.876) | (0.539-0.827) | (0.575-0.885) | (0.548-0.838) | (0.545-0.834) | (0.537-0.825) | (0.579-0.891) | (0.548-0.839) | (0.544-0.831) |
| **Social participation**  **(Yes)** | 0.725^**^ |  |  |  |  |  |  |  |  |  |
|  | (0.586-0.896) |  |  |  |  |  |  |  |  |  |
| **Levels of**  **Social participation** |  | 0.919 |  |  |  |  |  |  |  |  |
|  |  | (0.840-1.006) |  |  |  |  |  |  |  |  |
| **Voluntary activities (Yes)** |  |  | 1.056 |  |  |  |  |  |  |  |
|  |  |  | (0.831-1.342) |  |  |  |  |  |  |  |
| **Recreation (Yes)** |  |  |  | 0.719^**^ |  |  |  |  |  |  |
|  |  |  |  | (0.583-0.887) |  |  |  |  |  |  |
| **Cultural activities**  **(Yes)** |  |  |  |  | 0.873 |  |  |  |  |  |
|  |  |  |  |  | (0.549-1.388) |  |  |  |  |  |
| **Other activities**  **(Yes)** |  |  |  |  |  | 0.909 |  |  |  |  |
|  |  |  |  |  |  | (0.499-1.655) |  |  |  |  |
| **Levels of voluntary activities** | | |  |  |  |  |  |  |  |  |
| One |  |  |  |  |  |  | 1.005 |  |  |  |
|  |  |  |  |  |  |  | (0.778-1.300) |  |  |  |
| Two |  |  |  |  |  |  | 1.471 |  |  |  |
|  |  |  |  |  |  |  | (0.868-2.491) |  |  |  |
| Three |  |  |  |  |  |  | 0.701 |  |  |  |
|  |  |  |  |  |  |  | (0.148-3.321) |  |  |  |
| **Levels of recreation** | |  |  |  |  |  |  |  |  |  |
| One |  |  |  |  |  |  |  | 0.755^*^ |  |  |
|  |  |  |  |  |  |  |  | (0.601-0.949) |  |  |
| Two |  |  |  |  |  |  |  | 0.635^**^ |  |  |
|  |  |  |  |  |  |  |  | (0.459-0.880) |  |  |
| Three |  |  |  |  |  |  |  | 0.656 |  |  |
|  |  |  |  |  |  |  |  | (0.279-1.541) |  |  |
| **Levels of Cultural activities** | | |  |  |  |  |  |  |  |  |
| One |  |  |  |  |  |  |  |  | 0.893 |  |
|  |  |  |  |  |  |  |  |  | (0.552-1.446) |  |
| Two |  |  |  |  |  |  |  |  | 0.685 |  |
|  |  |  |  |  |  |  |  |  | (0.140-3.343) |  |
| **Levels of other activities** | |  |  |  |  |  |  |  |  |  |
| One |  |  |  |  |  |  |  |  |  | 0.848 |
|  |  |  |  |  |  |  |  |  |  | (0.459-1.568) |
| Two |  |  |  |  |  |  |  |  |  | - |
|  |  |  |  |  |  |  |  |  |  | - |
| **Constant** | 262.417^***^ | 232.850^***^ | 229.345^***^ | 242.800^***^ | 226.981^***^ | 231.285^***^ | 230.763^***^ | 238.040^***^ | 228.093^***^ | 228.888^***^ |
|  | (41.815-1646.832) | (37.204-1457.325) | (36.596-1437.280) | (38.616-1526.613) | (36.220-1422.437) | (36.940-1448.091) | (36.815-1446.450) | (37.806-1498.807) | (36.390-1429.696) | (36.546-1433.516) |
| N | 2452 | 2452 | 2452 | 2452 | 2452 | 2452 | 2452 | 2452 | 2452 | 2452 |

*Control variables were included in all models; ^*^ p < 0.05, ^**^ p < 0.01, ^***^ p < 0.001*

**S10** Regression results for social participation and depression levels in men

| **Variable** | (1) | (2) | (3) | (4) | (5) | (6) | (7) | (8) | (9) | (10) |
| --- | --- | --- | --- | --- | --- | --- | --- | --- | --- | --- |
| **WeChat usage** | -0.504^*^ | -0.525^**^ | -0.595^**^ | -0.504^*^ | -0.593^**^ | -0.594^**^ | -0.599^**^ | -0.495^*^ | -0.597^**^ | -0.596^**^ |
|  | (0.199) | (0.201) | (0.198) | (0.199) | (0.196) | (0.196) | (0.198) | (0.199) | (0.197) | (0.196) |
| **Social participation**  **(Yes)** | -0.498^*^ |  |  |  |  |  |  |  |  |  |
|  | (0.203) |  |  |  |  |  |  |  |  |  |
| **Levels of**  **Social participation** |  | -0.122 |  |  |  |  |  |  |  |  |
|  |  | (0.081) |  |  |  |  |  |  |  |  |
| **Voluntary activities (Yes)** |  |  | -0.019 |  |  |  |  |  |  |  |
|  |  |  | (0.226) |  |  |  |  |  |  |  |
| **Recreation (Yes)** |  |  |  | -0.497^*^ |  |  |  |  |  |  |
|  |  |  |  | (0.199) |  |  |  |  |  |  |
| **Cultural activities**  **(Yes)** |  |  |  |  | -0.081 |  |  |  |  |  |
|  |  |  |  |  | (0.408) |  |  |  |  |  |
| **Other activities**  **(Yes)** |  |  |  |  |  | -0.123 |  |  |  |  |
|  |  |  |  |  |  | (0.516) |  |  |  |  |
| **Levels of voluntary activities** | | |  |  |  |  |  |  |  |  |
| One |  |  |  |  |  |  | -0.111 |  |  |  |
|  |  |  |  |  |  |  | (0.242) |  |  |  |
| Two |  |  |  |  |  |  | 0.581 |  |  |  |
|  |  |  |  |  |  |  | (0.516) |  |  |  |
| Three |  |  |  |  |  |  | -0.511 |  |  |  |
|  |  |  |  |  |  |  | (1.277) |  |  |  |
| **Levels of recreation** | |  |  |  |  |  |  |  |  |  |
| One |  |  |  |  |  |  |  | -0.435^*^ |  |  |
|  |  |  |  |  |  |  |  | (0.217) |  |  |
| Two |  |  |  |  |  |  |  | -0.632^*^ |  |  |
|  |  |  |  |  |  |  |  | (0.288) |  |  |
| Three |  |  |  |  |  |  |  | -0.690 |  |  |
|  |  |  |  |  |  |  |  | (0.715) |  |  |
| **Levels of Cultural activities** | | |  |  |  |  |  |  |  |  |
| One |  |  |  |  |  |  |  |  | -0.172 |  |
|  |  |  |  |  |  |  |  |  | (0.429) |  |
| Two |  |  |  |  |  |  |  |  | 0.686 |  |
|  |  |  |  |  |  |  |  |  | (1.195) |  |
| **Levels of other activities** | |  |  |  |  |  |  |  |  |  |
| One |  |  |  |  |  |  |  |  |  | -0.166 |
|  |  |  |  |  |  |  |  |  |  | (0.519) |
| Two |  |  |  |  |  |  |  |  |  | 3.845 |
|  |  |  |  |  |  |  |  |  |  | (4.746) |
| **Constant** | 26.696^***^ | 26.501^***^ | 26.528^***^ | 26.579^***^ | 26.511^***^ | 26.516^***^ | 26.546^***^ | 26.534^***^ | 26.494^***^ | 26.507^***^ |
|  | (1.825) | (1.825) | (1.827) | (1.824) | (1.827) | (1.826) | (1.827) | (1.825) | (1.828) | (1.826) |
| N | 2452 | 2452 | 2452 | 2452 | 2452 | 2452 | 2452 | 2452 | 2452 | 2452 |

*Standard errors in parentheses; Control variables were included in all models; ^*^ p < 0.05, ^**^ p < 0.01, ^***^ p < 0.001*

**S11** KHB test in the middle-aged

| **Effect** | **β** | **SE** | **P** | **95% CI** | |
| --- | --- | --- | --- | --- | --- |
|  |  |  |  | **Lower** | **Upper** |
| **WeChat usage--Social participation--Depression** | | | | | |
| Total effect | -0.302 | 0.087 | 0.001 | -0.473 | -0.132 |
| Direct effect | -0.274 | 0.088 | 0.002 | -0.447 | -0.102 |
| Indirect effect | -0.028 | 0.014 | 0.050 | -0.056 | 0.000 |
| **WeChat usage--Levels of social participation--Depression** | | | | | |
| Total effect | -0.304 | 0.087 | 0.000 | -0.474 | -0.133 |
| Direct effect | -0.264 | 0.089 | 0.003 | -0.438 | -0.090 |
| Indirect effect | -0.040 | 0.019 | 0.038 | -0.077 | -0.002 |
| **WeChat usage--Recreation--Depression** | | | | | |
| Total effect | -0.302 | 0.087 | 0.001 | -0.473 | -0.132 |
| Direct effect | -0.271 | 0.088 | 0.002 | -0.444 | -0.098 |
| Indirect effect | -0.032 | 0.015 | 0.036 | -0.061 | -0.002 |
| **WeChat usage--Levels of recreation--Depression** | | | | | |
| Total effect | -0.304 | 0.087 | 0.000 | -0.474 | -0.133 |
| Direct effect | -0.256 | 0.088 | 0.004 | -0.429 | -0.083 |
| Indirect effect | -0.048 | 0.017 | 0.006 | -0.082 | -0.014 |
| **WeChat usage--Levels of social participation--Levels of depression** | | | | | |
| Total effect | -0.650 | 0.182 | 0.000 | -1.006 | -0.294 |
| Direct effect | -0.575 | 0.186 | 0.002 | -0.939 | -0.212 |
| Indirect effect | -0.074 | 0.038 | 0.053 | -0.150 | 0.001 |

**S12** KHB test in the elderly

| **Effect** | **β** | **SE** | **P** | **95% CI** | |
| --- | --- | --- | --- | --- | --- |
|  |  |  |  | **Lower** | **Upper** |
| **WeChat usage--Social participation--Depression** | | | | | |
| Total effect | -0.583 | 0.159 | 0.000 | -0.895 | -0.271 |
| Direct effect | -0.506 | 0.161 | 0.002 | -0.823 | -0.190 |
| Indirect effect | -0.077 | 0.030 | 0.011 | -0.136 | -0.017 |
| **WeChat usage--Recreation--Depression** | | | | | |
| Total effect | -0.582 | 0.159 | 0.000 | -0.894 | -0.271 |
| Direct effect | -0.529 | 0.161 | 0.001 | -0.844 | -0.214 |
| Indirect effect | -0.053 | 0.028 | 0.055 | -0.108 | 0.001 |
| **WeChat usage-- social participation--Levels of depression** | | | | | |
| Total effect | -1.165 | 0.280 | 0.000 | -1.714 | -0.615 |
| Direct effect | -1.019 | 0.285 | 0.000 | -1.578 | -0.460 |
| Indirect effect | -0.146 | 0.058 | 0.012 | -0.259 | -0.033 |
| **WeChat usage--Levels of social participation--Levels of depression** | | | | | |
| Total effect | -1.165 | 0.281 | 0.000 | -1.714 | -0.615 |
| Direct effect | -1.014 | 0.290 | 0.000 | -1.583 | -0.445 |
| Indirect effect | -0.150 | 0.076 | 0.048 | -0.299 | -0.002 |
| **WeChat usage--Levels of voluntary activities--Levels of depression** | | | | | |
| Total effect | -1.165 | 0.281 | 0.000 | -1.715 | -0.614 |
| Direct effect | -1.084 | 0.287 | 0.000 | -1.646 | -0.522 |
| Indirect effect | -0.081 | 0.060 | 0.180 | -0.199 | 0.037 |
| **WeChat usage--Levels of recreation--Levels of depression** | | | | | |
| Total effect | -1.165 | 0.281 | 0.000 | -1.715 | -0.615 |
| Direct effect | -1.049 | 0.286 | 0.000 | -1.610 | -0.488 |
| Indirect effect | -0.116 | 0.058 | 0.048 | -0.230 | -0.001 |

**S13** KHB test in men

| **Effect** | **β** | **SE** | **P** | **95% CI** | |
| --- | --- | --- | --- | --- | --- |
|  |  |  |  | **Lower** | **Upper** |
| **WeChat usage--Social participation--Depression** | | | | | |
| Total effect | -0.400 | 0.108 | 0.000 | -0.612 | -0.187 |
| Direct effect | -0.339 | 0.110 | 0.002 | -0.554 | -0.123 |
| Indirect effect | -0.061 | 0.021 | 0.004 | -0.103 | -0.019 |
| **WeChat usage--Recreation--Depression** | | | | | |
| Total effect | -0.400 | 0.108 | 0.000 | -0.612 | -0.187 |
| Direct effect | -0.338 | 0.110 | 0.002 | -0.553 | -0.123 |
| Indirect effect | -0.062 | 0.021 | 0.003 | -0.103 | -0.020 |
| **WeChat usage--Levels of recreation--Depression** | | | | | |
| Total effect | -0.401 | 0.108 | 0.000 | -0.613 | -0.188 |
| Direct effect | -0.331 | 0.110 | 0.003 | -0.547 | -0.115 |
| Indirect effect | -0.070 | 0.023 | 0.002 | -0.115 | -0.025 |
| **WeChat usage--Social participation--Levels of depression** | | | | | |
| Total effect | -0.598 | 0.195 | 0.002 | -0.980 | -0.215 |
| Direct effect | -0.504 | 0.199 | 0.011 | -0.893 | -0.114 |
| Indirect effect | -0.094 | 0.040 | 0.017 | -0.171 | -0.016 |
| **WeChat usage--Recreation--Levels of depression** | | | | | |
| Total effect | -0.598 | 0.195 | 0.002 | -0.980 | -0.215 |
| Direct effect | -0.504 | 0.199 | 0.011 | -0.894 | -0.115 |
| Indirect effect | -0.093 | 0.039 | 0.016 | -0.169 | -0.018 |
| **WeChat usage--Levels of recreation--Levels of depression** | | | | | |
| Total effect | -0.598 | 0.195 | 0.002 | -0.980 | -0.215 |
| Direct effect | -0.495 | 0.199 | 0.013 | -0.885 | -0.105 |
| Indirect effect | -0.103 | 0.041 | 0.012 | -0.183 | -0.022 |
